# Effectiveness of Virtual Reality for Pain Relief in Procedures Related to Obstetrics and Gynaecology: A Systematic Review and Meta‐Analysis of Randomised Controlled Trials

**DOI:** 10.1111/1471-0528.70194

**Published:** 2026-02-24

**Authors:** Jhia Jiat Teh, Filipa Campos, Adam Koczoski, Trisha Valencia, Michael P. Rimmer, Bassel H. Al Wattar

**Affiliations:** ^1^ St Mary's Hospital Imperial College Healthcare NHS Trust London UK; ^2^ Faculty of Medicine, Department of Metabolism, Digestion and Reproduction Imperial College London London UK; ^3^ University of Cambridge Cambridge UK; ^4^ Centre for Reproductive Health, Institute of Regeneration and Repair, Edinburgh BioQuarter University of Edinburgh Edinburgh UK; ^5^ Edinburgh Fertility Centre Royal Infirmary of Edinburgh Edinburgh UK; ^6^ University College London London UK; ^7^ Beginnings Assisted Conception Unit Epsom and St Helier University Hospitals London UK

**Keywords:** analgesia, gynaecology, obstetrics, pain, systematic review, virtual reality

## Abstract

**Background:**

Immersive virtual reality (VR) technology offers a non‐invasive, non‐pharmacological approach to reduce pain perception in patients undergoing diagnostic or interventional procedures.

**Objective:**

To systematically evaluate the efficacy of immersive VR technology in reducing pain perception during obstetric and gynaecological procedures.

**Search Strategy:**

We searched MEDLINE, EMBASE, CENTRAL, and CINAHL databases from inception to January 2025.

**Selection Criteria:**

We included randomised controlled trials (RCTs) evaluating VR interventions in women undergoing obstetric or gynaecological procedures.

**Data Collection and Analysis:**

We performed meta‐analyses using random‐effects models and assessed risk of bias using the Cochrane risk‐of‐bias tool for randomised trials.

**Main Results:**

49 RCTs (5355 participants) were included. Due to clinical heterogeneity, data were analysed separately. VR resulted in a larger, consistent reduction in pain scores compared with standard care in labour (11 studies; SMD −0.93, 95% CI −1.25 to −0.60) compared to minor procedures (29 studies; SMD −0.64, 95% CI −0.97 to −0.32). VR also significantly reduced anxiety scores in both labour (8 studies; SMD −1.13, 95% CI −1.80 to −0.45) and minor procedures (17 studies; SMD −0.74, 95% CI −1.18 to −0.29). Our analysis was limited by high levels of heterogeneity and variability in procedural protocols.

**Conclusion:**

Immersive VR technology appears effective for reducing pain and anxiety during obstetric and gynaecological procedures, particularly during childbirth, despite substantial statistical heterogeneity. Further research is needed to optimise implementation strategies and establish clinical practice guidelines.

## 1 Introduction

Pain constitutes the most ubiquitous clinical symptom, often as a result of medical procedures [1]. Ensuring effective pain management is pivotal to optimise procedural outcome, mitigate anxiety, enhance patient satisfaction and reduce use of analgesia [2]. Non‐pharmacological approaches to address pain and anxiety have gained significant attention, with the use of virtual reality (VR) technology emerging as a distraction technique for nonpharmacological pain relief [3]. VR technology is a relatively recent addition to healthcare interventions, involving the use of computer‐generated simulations typically viewed through a headset. This technology creates a highly immersive, interactive and multisensory virtual environment for users. The perception of presence and immersiveness are regarded as pivotal elements contributing to the therapeutic value and efficacy of VR as analgesia. VR alters the processing of nociceptive stimuli by stimulating the visual cortex [4]. Functional magnetic resonance imaging (fMRI) studies have shown that VR produces similar effects to the sensory cortex as opioids do, suggesting its potential as a nonpharmacological analgesic [5]. In the medical field, VR technology has found application in several areas such as surgical training, patient education, rehabilitation, and the management of pain and anxiety across numerous clinical settings. In recent years, there has been a substantial improvement in the cost, quality and accessibility of VR devices, which introduces innovative prospects for management of pain and anxiety across different medical disciplines [6].

VR for managing pain and anxiety has been extensively studied in paediatrics, dentistry, burns management and even in labour. Multiple meta‐analyses have suggested that VR may play a role in reducing pain scores in acutely painful procedures only, such as those involving needles and burns in physical therapy. These analyses were either limited to specific clinical situations, such as dressing changes or phlebotomy, or lacked comprehensive subgroup analyses to investigate the underlying reasons for heterogeneity [3]. Although there have been numerous studies in recent years evaluating the use of VR to ameliorate patients' pain or anxiety across a range of acute procedures, the applicability of VR technology within obstetrics and gynaecology is still in its infancy. A survey conducted by Harper and colleagues found wide acceptability for VR within obstetrics and gynaecology [7].

In obstetrics, the experience of labour varied significantly, with a high proportion of nulliparous women describing severe childbirth pain [8]. Although neuraxial blockade is commonly used for pain control in labouring women, there is increased interest in exploring options for managing labour pain, from pharmacotherapies, patient‐controlled analgesia, and nitrous oxide to non‐pharmacological methods including hydrotherapy, music therapy, acupuncture and transcutaneous electronic nerve stimulation [9, 10]. The benefits of non‐pharmacological pain relief have been reported including increased self‐confidence and satisfaction with birth experience, increased breastfeeding rates and mother‐infant attachment [11]. In gynaecology, there has been a significant move toward performing diagnostic and therapeutic procedures for gynaecological conditions in an outpatient setting. One example of which is outpatient hysteroscopy, which has advantages such as mitigating the risk associated with general anaesthesia, reducing healthcare expenditures and increasing convenience for patients. While these procedures are typically well‐tolerated, they can elicit acute pain and anxiety. The use of VR technology as a distraction technique has been studied across outpatient gynaecological procedures and in labour but the true efficacy remains unknown to underpin practice.

The objective of this study was to undertake a systematic review and meta‐analysis to compare VR efficacy as a non‐invasive and non‐pharmacological pain management method in patients undergoing medical procedures within obstetrics and gynaecology.

## 2 Methods

We conducted a systematic review and meta‐analysis using a prospectively registered protocol (CRD 42023394672) and reported our findings in accordance with PRISMA guidelines [12].

## 3 Data Sources and Search Strategy

We searched the following databases (MEDLINE, EMBASE, Cochrane Central Register of Controlled Trials (CENTRAL), CINAHL (Cumulative Index to Nursing and Allied Health Literature)) for randomised trials evaluating the effectiveness of immersive VR technology for pain relief or anxiety relief during any acute procedures in obstetrics and gynaecology. The search includes studies published from inception to 30th November 2025 using a combination of medical subject heading (MeSH) terms, keywords and word variants for ‘virtual reality’, ‘pain’, ‘anxiety’, ‘analgesia’, ‘fear’, ‘obstetrics’, ‘gynaecology’, ‘labour’, ‘hysteroscopy’, ‘colposcopy’, ‘episiotomy’, ‘external cephalic version’, ‘caesarean section’, ‘embryo transfer’, ‘amniocentesis’, ‘cystoscopy’ and combined them using Boolean operators (AND, OR) to conduct our searches and adjusted the strategy for each database (Appendix S1). To maximise sensitivity, we applied the Cochrane Highly Sensitive Search Strategy filters for identifying randomised controlled trials. No search filters or language limitations were employed; articles in non‐English languages had their abstract translated and if deemed relevant the whole article was translated for consideration for inclusion. We manually screened bibliographies of potentially relevant articles and published systematic reviews on the topic to identify any additional relevant trials.

## 4 Study Selection

Studies were eligible for inclusion if they were randomised trials of any design that evaluated the efficacy of any immersive VR technology equipment for pain relief or anxiety relief compared to standard care (including placebo or routine analgesia) during any acute procedures in obstetrics and gynaecology, including labour and childbirth. Non‐randomised studies, review articles, and animal studies were excluded. Additionally, studies that solely evaluated distraction techniques (utilising a display screen without immersive capabilities), those comparing VR directly against active pharmacological agents without a standard care control, and those that did not measure pain using a standardised tool or reported on pain scores more than an hour post‐procedure were also excluded.

We performed the study selection and data extraction processes in triplicate (AK, TV, FC), further checked by a fourth reviewer (JJT). Any discrepancies or disagreements among reviewers were discussed and resolved through consensus with two additional reviewers (MPR and BHW).

## 5 Data Extraction

We collected data on the country of study, publication journal, intervention settings, population characteristics, inclusion and exclusion criteria, type of VR technology and equipment used, nature of the medical procedure or intervention, duration of procedure, pain and/or anxiety scores used and adverse effects. Our primary outcome was pain scores measured immediately after or within an hour of the procedure. We also collected data on anxiety scores where relevant. In studies where pain and/or anxiety scores were measured at different time points, we recorded the values measured closest to the end of the procedure. In studies involving labour and childbirth, we recorded all available scores at different cervical dilation as reported by different studies.

## 6 Assessment of Risk of Bias

Three independent reviewers (AK, TV, and FC) assessed the quality of included studies using the Cochrane Risk of Bias assessment tool 2. Each study was assessed for the quality of randomisation and sequence generation, allocation to intervention groups, outcome assessment, completeness of outcome data, and selective outcome reporting. Due to the nature of the intervention, we did not penalise unblinded trials. Each domain was scored as low, unclear, or high risk of bias. In case of uncertainty, consensus was established with input from a third reviewer (JJT).

## 7 Data Synthesis

Statistical analyses were performed using R (version 4.5.2) with the meta and metafor packages. Continuous outcomes (pain and anxiety scores) were analysed using the Standardised Mean Difference (SMD) with 95% confidence intervals (CI) to account for variations in measurement scales (e.g., VAS 0–10 vs. NRS 0–100). Random‐effects models were employed for all analyses using the Restricted Maximum Likelihood (REML) method, as we anticipated clinical heterogeneity across the diverse range of procedures.

Heterogeneity was quantified using the *I* ^2^ statistics. The *I* ^2^ index is a more recent approach to quantify heterogeneity in meta‐analyses [13]. *I* ^2^ provides an estimate of the percentage of variability in results across studies that is due to real differences and not due to chance [13]. The *I* ^2^ index measures the extent of heterogeneity by dividing the result of Cochran's Q test and its degrees of freedom by the *Q*‐value itself. An *I* ^2^ of less than 25% is usually viewed as low heterogeneity, between 25% and 50% as moderate, and over 50% as high heterogeneity [13].

Given the distinct clinical nature of labour pain versus acute procedural pain, we performed a pre‐specified subgroup analysis stratifying studies into two broad categories: (1) Labour and (2) Minor Gynaecological Procedures. To further explore heterogeneity within these groups, we conducted secondary subgroup analyses: (i) Labour: Stratified by stage (Early vs. Late/Transitional phase). (ii) Minor Procedures: Stratified by procedure type (Hysteroscopy, IUD insertion, Episiotomy repair, and Other). In accordance with recent statistical recommendations for meta‐analyses with high heterogeneity, we calculated 95% prediction intervals. Unlike confidence intervals, which estimate the mean effect, prediction intervals estimate the range of effect sizes expected in future individual studies, providing a more clinically relevant measure of consistency. We also assessed the publication bias and small study effect using a funnel plot for each pairwise comparison and performed Egger's test to assess its statistical significance.

## 8 Results

### 8.1 Summary of Study Characteristics

**FIGURE 1:**
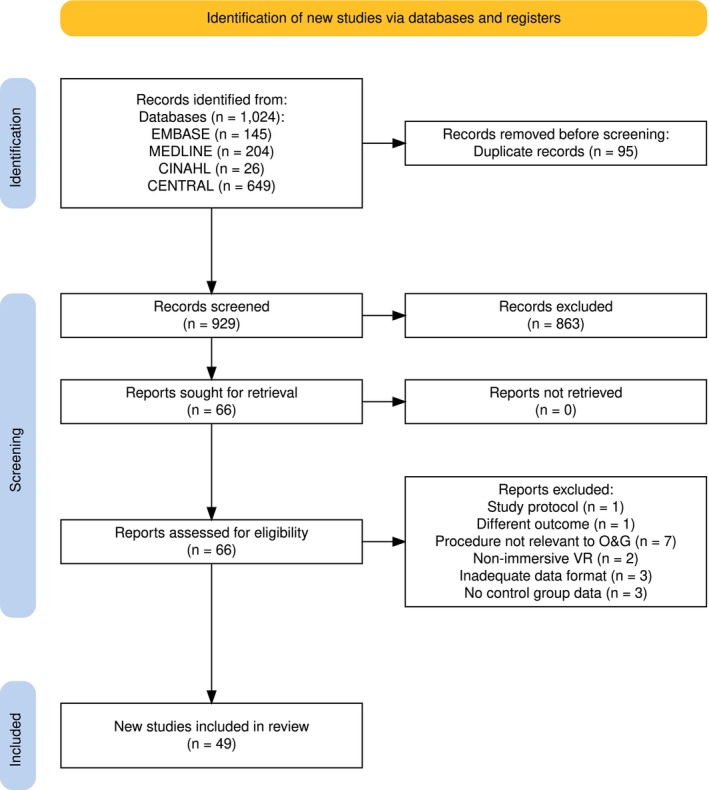
PRISMA flow chart of included studies evaluating the effectiveness of virtual reality for pain control and anxiety in procedures relating to obstetrics and gynaecology.

The electronic search yielded 1024 potentially relevant citations of which we screened 929 in full and included 49 in our meta‐analysis (Figure 1). No relevant citations were identified in non‐English. All included RCTs had a minimum of two‐group parallel design comparing VR against standard care (control). The most common reasons for exclusion of studies were failure to report the outcome or the exposure of interest, study designs that were not included in the eligibility criteria, and non‐original study reports (i.e., letters, reviews, or editorials) (Appendices S2 and S3). Of these 49 studies, 40 had data available on the effect of VR on pain and 25 had data available on the effect of VR on anxiety, and 20 had data available on both outcomes of interest. The included studies represented populations from diverse regions. Twenty studies were based in the Middle East and North Africa (Turkey, Iran, Saudi Arabia, Israel, Egypt). Fourteen studies represented Europe (UK, Germany, Belgium, France, Netherlands, Spain, Italy, Ireland). Seven studies were from North America (Mexico, USA, Canada), while the remainder were from Oceania (Australia, New Zealand), Southeast/East Asia (China, Thailand) and South Asia (India) (Table 1 and Table S1).

**TABLE 1:** Description of the VR equipment and software used in randomised trials evaluating the effectiveness of virtual reality for pain control in medical procedures.

Study	Country	VR equipment	VR software/Environment	Interactive or non‐interactive	Age (mean ± SD)/median (IQR)
Akin 2021	Turkey	VR Box 3D virtual reality glass	Video images recorded on the phone of the pregnant woman by looking at the baby's face with the help of a 3D/4D probe	Non‐interactive	O: 27.23 ± 3.1
Almedhesh 2022	Saudi Arabia	Oculus Rift S PC‐powered VR headset	Landscape with calm music or Holy Quran with landscapes	Non‐interactive	I: 32.2 C: 32.28
Bal 2025	Turkey	VR headset with smartphone (model not specified)	30‐min video of natural settings (plants, land, water, sky) with nature sounds	Non‐interactive	I: 29 (median), C: 30 (median)
Baltaci 2024	Turkey	Not specified	Relaxing video with nature content	Non‐interactive	I: 29.6 ± 6.1 C: 28.6 ± 4.2
Benazzouz 2023	France	Not specified	Choice of four virtual universes: forest, the meadow, the ocean floor, or outer space	Non‐interactive	I: 23.8 ± 4.9 C: 23.5 ± 4.6
Boyuk 2025	Turkey	Oculus Go (Meta/Xiaomi)	20‐min nature videos (snow, forest, rainforest, beach)	Non‐interactive	I: 25.4 ± 3.4 C: 26.1 ± 4.3
Brunn 2022	USA	Oculus Go headset with guided meditation VR App	Nokia Spot 1 environment and 10‐min guided Zen meditation	Interactive	I: 37 ± 4.2 C: 37 ± 5.2 O: 37 ± 4.7
Carus 2022	Turkey	Oculus Quest All‐in‐one VR Gaming Headset (128 GB) VR system	Several virtual environments, including orange sunset, green meadows, black beginning, red savannah, blue deep, blue moon, blue ocean, white winter, and red fall	Non‐interactive	I: 31.0 ± 2.6 C:31.8 ± 3.6
Chinanuwatwong 2025	Thailand	VR Headset (Adapted)	Video of scenic landscapes with relaxing instrumental music	Non‐interactive	I: 33.3 ± 6.4 C: 32.1 ± 5.7
Cowles 2019	USA	Not reported	Not reported	Not reported	Not reported
Deo 2021	UK	Oculus Go with a head mounted display and built‐in audio drivers	8‐min video called ‘Forest of Serenity’ commissioned by St Giles Hospice, developed by Holosphere and narrated by Sir David Attenborough	Interactive	I:31.0 ± 2.6 C:31.8 ± 3.6
Dumont 2025	Australia	Smartphone and Headset (Smileyscope)	3D video of seals swimming with gentle classical music	Non‐interactive	I: 33.5 ± 9.0 C: 33.0 ± 9.2
Dviri 2020	Canada	“Oculus Rift” system	Three different calming VR environments (3 scenes: beach sunset, palm tree patio or redwood forest)	Non‐interactive	Not reported
Ebrahimian 2022	Iran	Virtual reality glasses (Samsung Gear VR Virtual Reality Headset with Samsung Mobile S7)	360‐degree video with nature landscapes	Non‐interactive	Not reported
Estrella‐Juarez 2022	Spain	Bnext 3D glasses with compatible smartphone	Images of the ocean floor with relaxing sounds	Non‐interactive	I: 31.1 (4.52), C:31.6 (5.16)
Fouks 2022	Israel	Head‐mounted display (SootheVR: AppliedVR, Los Angeles, California)	Immersive module of diving in a lagoon	Interactive	I: 39 (32–51.2) C: 38 (34–48.8)
Frey 2018	USA	Samsung Gear Oculus VR headset fitted with Samsung Galaxy S5 Note phone.	Non‐interactive animation Cartoon videos.	Non‐interactive	O: 27.9 ± 5.6
Gamal 2025	Egypt	VR Headset (model unspecified)	“Virtual Nature 360” video (waterfalls and beaches)	Non‐interactive	I: 44 (29–54) C: 41 (36–48)
Gur 2020	Turkey	Samsung Gear VR2 VRG	Video and digital photograph album composed of photographs of healthy, not crying, and calm newborn or classical music (Beethoven‐Moonlight sonata) or introductory film of Turkey	Non‐interactive	Ia: 25.6 ± 5.1 Ib: 25.9 ± 4.3 Ic: 25.4 ± 4.5 Id: 27.7 ± 6.4 C: 26.4 ± 4.3
Hecken 2023	Germany	VR headset (Pico G2 4K; Qingdao Pico Technology Co Ltd., China) and an Android tablet for remote control	2 nature sceneries and “fairy tale wood”, a cartoon, and 2 features (tropical forest, underwater swimming with dolphins) for the “during colposcopy” exposure	Non‐interactive	C: 44.14 ± 11.32 Ia: 43.97 ± 9.66 Ib: 43.9 ± 11.24
Higgins 2025	Belgium	Oculus Go (Meta Reality Labs)	Nature setting with soothing sounds	Non‐interactive	I: 36 ± 7 C: 34 ± 6
Jahani Shoorab 2015	Iran	VR glasses playing 3D film with two external headphones (3D blue‐ray/DVD player full HD).	Non‐interactive videos (Dolpine and Whales)	Non‐interactive	Ol: 24.1 ± 4.1
Keles 2025	Turkey	Everest VR‐0023 3D glasses with smartphone	Nature video (“Relaxation Project 1” from YouTube) accompanied by Acemashiran music	Non‐interactive	I: 22.3 ± 3.5 C: 21.2 ± 2.7
Kirca 2023	Turkey	Not specified	Not specified	Non‐interactive	I: 23.5 ± 3.4 C: 22.6 ± 4.1
Kleiner 2023	USA	Not specified	Not specified	Non‐interactive	Not reported
Mahalan 2023	India	Procus ONE Virtual reality headset 40 mm lenses‐For ‘IOS and Android’, a head‐mounted display powered by a Galaxy J7 prime, two phones, and headphones	Slideshow of images of pregnant women or breastfeeding mothers and playing Raga Desi Todi for an hour in two 30‐min cycles (20 min intervention +10 min break)	Non‐interactiveon	I: 23.1 ± 3.8 C: 23.5 ± 3.7
Massov 2022	New Zealand	VR Oculus Go	Scene of playful dolphins from the Ocean Rift scuba diving simulation, with accompanying dolphin sounds, breathing sounds and an overture of classical music	Non‐interactive	O: 71.4% were between 25 and 34, 28.5% over 34
McDougall 2024	UK	Stand‐alone VR headset, Oculus Go with a head‐mounted display and in‐built audio drivers	Guided relaxation experience ‘The Forest of Serenity’	Non‐interactive	I: 32 ± 6.9 C: 33 ± 5.1
Melcer 2021	Israel	Oculus Go standalone VR headset	5–15‐min clip showing views of rolling hills, sail boats, a tropical beach, a beautiful desert landscape and the undersea world	Non‐interactive	I: 34 ± 4.9 C: 36.7 ± 3.3
Mohammadi 2023	Iran	Samsung Gear VR Headset	Game containing a pleasant sound (flow of water) simulating the sea shore	Interactive	I: 44.6% 21–25 C: 38.5% 21–25
Momenyan 2021	Iran	Android application was developed using the Google VR SDK, patient's head movements tracked using Samsung S3's inertial measurement unit (IMU) sensor	360 degrees video of nature containing beach and peaceful landscape along with the sound of nature	Non‐interactive	I: 28.41 (4.50) C: 30.37 (6.09) O: 29.39 (5.39)
Ng 2025	Hong Kong	Oculus Quest 2	4‐min loop of forest/lake scenery with nature sounds	Non‐interactive	I: 35.9 ± 3.0 C: 35.9 ± 2.5
Olloqui 2025	Spain	Oculus Quest 2 headset (Meta Platforms)	360‐degree video of a gondola ride in Venice with relaxing music (HealthNap Sedación Digital)	Non‐interactive	I: 44.2 ± 13.0 C: 46.2 ± 10.7
Oz 2024a	Turkey	Everest VR0022 VR BOX virtual reality glasses	Nature walk accompanied by music	Non‐interactive	I: 33.23 ± 6.0 C: 35.5 ± 4.8
Oz 2024b	Turkey	Not specified	Forest view or sea view	Non‐interactive	I: 36.3 ± 7.5 C: 38.4 ± 9.3
Pelazas‐Hernandez 2023	Spain	Samsung's Oculus Go model	A Night Sky, where patient is invited to relax on edge of peaceful vale, beside a crackling campfire and beneath the open night sky—connecting constellation to form patterns among the stars using a device	Interactive	I: 47.2 ± 8.7 C: 49.2 ± 11.8
Rosielle 2024	Netherlands	The head‐mounted PICO G2 4K device (Pico Interactive Inc., San Francisco, USA)	SyncVR Relax and Distract, 20 relaxing movies and breathing exercises	Interactive	I: 35 ± 5 C: 35 ± 4
Schutyser 2021	Belgium	Head mounted smartphone with headphones	Oncomfort, autohypnosis environment	Interactive	O: 18–48
Sewell 2023	UK	Pico G2 VR headset and Hypno VR software	Four seasons zen environment	Non‐interactive	I: 47.8 ± 11.3 C: 49.6 ± 11.3
Sezer 2023	Turkey	VR SHINECON	Video of the sea, underwater, and forest through VR glasses. Patients also listened to relaxing music	Non‐interactive	I: 30.4 ± 6.4 C: 31.5 ± 5.6
Sibal 2025	Turkey	Efnan G04EA VR Shinecon 3D headset with smartphone	Pre‐recorded 360° nature scenery videos (YouTube) without audio/music	Non‐interactive	I: 48 ± 13 C: 56 ± 11
Smith 2020	Australia	HMD with a Samsung Galaxy S8 smartphone, head tracking, hand controllers and touchpad	Interactive game (Skylight) with relaxing background music is also played to provide auditory stimulation.	Interactive	I: 32.1 ± 5.5 C: 31.0 ± 5.5 O: 31.6 ± 5.5
Sunay 2025	Turkey	HTC VIVE Cosmos PC Virtual Reality goggles	Game‐based VR: Balloon inflating (breathing), balloon popping (squatting), gold collecting (swinging), plus relaxation nature scenes	Interactive	I: 25.4 ± 4.9 C: 21.8 ± 4.5
Tarriel 2025	Spain	Portable, standalone VR headset PICO G2 (Pico XR, Mountain View, CA) with a head‐mounted display with built‐in audio speaker	Prior to the hysteroscopy procedure, patients viewed a 7 min conscious and guided relaxation “body‐scan” procedure. Once the procedure began, watched “Under the Sea”, representing a video game‐like environment where patients were asked to look for specific sea life	Interactive	I: 43.3 ± 10 C: 45.6 ± 9.9
Toker 2025	Turkey	VR glasses	Relaxation video featuring nature scenes accompanied by calming music	Non‐interactive	I: 30.1 ± 6.8 C: 29.6 ± 5.5
Wong 2021	USA	VR google with imagery and auditory guidance	Non‐interactive videos of a blossoming tree, ocean waves, and crackling campfire with meditative auditory guidance.	Non‐interactive	I: 31.6 (5.6) C: 32.5 (3.6)
Xie 2022	China	3D SpaceMax software	The real scene shooting in the delivery room is added to the system to bring the 3D interactive virtual scene to life, including characters, sites, objects, environments, time and voices.	Non‐interactive	I: 30.3 ± 1.14 C: 30.4 ± 1.07
Xu 2024	China	Head‐mounted display of PICO Neo 3 (Pico Technology CO Ltd) 98° field of view	The first part was popular science topics, such as the caesarean section process and physical experiences after anaesthesia; the second part was landscape meditation, meditation lines	Non‐interactive	I: 34 ± 4 C: 34 ± 5
Zizlofi 2025	Italy	Deepsen VRx Device	Relaxing environments (mountain, hill, river) with audio‐guided breathing and music	Non‐interactive	I: 45 ± 13 C: 43.2 ± 11.3

Abbreviations: C, control; HMD, head mounted display; I, intervention; O, overall; PC, personal computer; VR, virtual reality.

Forty‐seven were parallel group studies and 2 were crossover studies, with 5355 patients in total. Twenty‐one studies were performed in an inpatient setting: 15 studied labour [14, 15, 16, 17, 18, 19, 20, 21, 22, 23, 24, 25, 26, 27, 28], 3 were conducted during episiotomy repair [29, 30, 31], 1 examined induction of labour [32], 2 studies concerned caesarean sections [33, 34], 1 was during external cephalic version [35]. The majority of the outpatient studies (*κ* = 27) were conducted during hysteroscopies (*κ* = 10) [36, 37, 38, 39, 40, 41, 42, 43, 44, 45], intrauterine device insertion (*κ* = 5) [46, 47, 48, 49, 50], hysterosalpingography (*κ* = 3) [51, 52, 53]. The remaining outpatient studies examined amniocentesis (*κ* = 2) [54, 55], colposcopy [56], embryo transfers [57], oocyte retrieval [58], intrauterine insemination [59] and urodynamics [60].

Outcomes included pain (40 studies), anxiety (25 studies), satisfaction (10 studies), stress (2 studies) and fear (2 studies). Outcome measures were heterogeneous, but the majority utilised either the Visual Analogue Scale (VAS) or a Numerical Rating Scale (NRS). With regards to the intervention, the majority of the reports (*n* = 35) used non‐interactive VR environments (e.g., passive videos of nature), while 14 studies utilised interactive VR environments. Forty studies reported using head‐mounted VR displays, with the remainder using other devices (e.g., smartphone‐based googles).

The majority of studies (*n* = 45) found no adverse effects associated with VR use. Where side effects were noted (e.g., nausea, dizziness), they were minor and did not differ significantly between intervention and control groups. The most common adverse effects reported included nausea, dizziness, vomiting, tremulousness, and flushing.

### 8.2 Quality Assessment of Included Studies

Majority of studies (29/49%, 59%) included in the meta‐analysis were assessed as having a low overall risk of bias. Approximately 10% (5/49) showed some concerns, and 31% (15/49) had a high overall risk of bias. Regarding the randomisation process, the majority of studies (76%) demonstrated low risk, though 11 studies showed some concerns due to unclear sequence generation. For adherence to the intervention, although participants and personnel were aware of the intervention due to the nature of VR, we judged the majority of trials (73%) as low risk because deviations from the intended intervention were minimal and reflected usual clinical practice. In the domain of outcome measurement, 9 trials showed some concerns; however, detection bias was generally considered low (82%) as valid, standardised scales (VAS/NRS) were utilised across studies. Reporting bias was minimal, with only 7 studies raising concerns or high risk. Two studies utilising a crossover design were also assessed for carryover effects, with one judged as low risk and the other as high risk (Table S2).

### 8.3 Publication Bias Assessment

Funnel plots and Egger's regression tests were conducted separately for the two clinical settings. Among studies of minor procedures (*n* = 29), visual inspection of the funnel plot suggested asymmetry, and Egger's regression test indicated the presence of small‐study effects (*t* = −2.58, df = 27, *p* = 0.016). Trim‐and‐fill analysis added seven imputed studies, resulting in an adjusted pooled effect that was attenuated toward the null (SMD = −0.24, 95% CI [−0.63, 0.16], *p* = 0.24), suggesting that the initial effect estimate may have been inflated by small‐study effects (Figure S1). Heterogeneity remained high (*I* ^2^ = 94.8%). In contrast, studies of labour (*n* = 11) showed no evidence of funnel plot asymmetry (Egger's test: *t* = −0.44, df = 9, *p* = 0.667). Given the smaller number of studies, trim‐and‐fill was not performed (Figure S2).

### 8.4 Synthesis of Results

#### 8.4.1 Pain

We meta‐analysed 40 studies (*n* = 3850) assessing pain scores. Due to significant clinical heterogeneity between labour pain and acute minor gynaecological procedures, these groups were analysed separately.

#### 8.4.2 Labour

**FIGURE 2:**
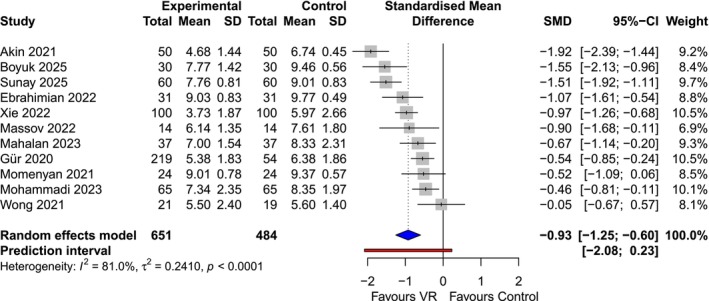
Forest plot showing the standardised mean difference (SMD) of VR versus control for pain score during labour. Individual studies are listed by first author and year. The size of each square is proportional to the study's weight in the random‐effects meta‐analysis. Horizontal lines indicate the 95% Confidence Interval (CI). The diamond represents the pooled effect estimate for all minor procedures. A negative SMD indicates a reduction in pain intensity favouring the VR group. The horizontal bar extending beyond the diamond represents the 95% prediction interval, estimating the range of effects expected in a future study. CI, confidence interval; SMD, standardised mean difference.

In the labour subgroup (*κ* = 11, *n* = 1135), VR intervention resulted in a large, statistically significant reduction in pain intensity compared to standard care (SMD −0.93; 95% CI: −1.25 to −0.60) (Figure 2). This effect size corresponds to an approximate reduction of 2.2 cm on a 10 cm Visual Analogue Scale (VAS). Heterogeneity was high (*I* ^2^ = 81%), likely due to variation in trial protocol and VR content. However, the 95% prediction interval was [−2.08 to 0.23], with the vast majority of the distribution indicating a beneficial effect.

Subgroup analysis by labour stage demonstrated consistent efficacy. The reduction in pain was significant during both the active phase of the first stage of labour (κ =10, *n* = 946, SMD −0.77; 95% CI: −1.25 to −0.29) and appeared more pronounced during the transitional phase (*κ* = 6, *n* = 658; SMD −1.15; 95% CI: −1.62 to −0.67), although the difference between subgroups was not statistically significant (*p* = 0.27) (Figure S3).

#### 8.4.3 Minor Procedures

**FIGURE 3:**
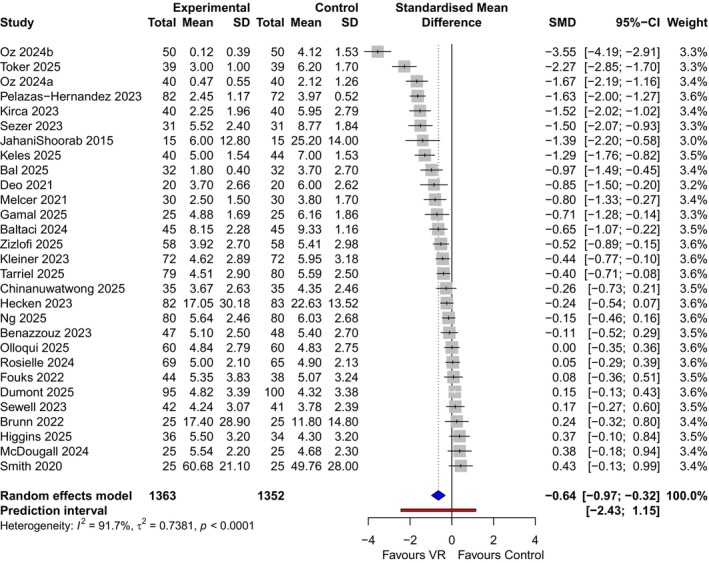
Forest plot showing the standardised mean difference (SMD) of VR versus control for pain score in minor procedures in obstetrics and gynaecology. Individual studies are listed by first author and year. The size of each square is proportional to the study's weight in the random‐effects meta‐analysis. Horizontal lines indicate the 95% Confidence Interval (CI). The diamond represents the pooled effect estimate for all minor procedures. A negative SMD indicates a reduction in pain intensity favouring the VR group. The horizontal bar extending beyond the diamond represents the 95% prediction interval, estimating the range of effects expected in a future study. CI, confidence interval; SMD, standardised mean difference.

For minor procedures (*κ* = 29, *n* = 2715), the pooled analysis showed a significant reduction in pain scores (SMD −0.64; 95% CI: −0.97 to −0.32) (Figure 3). However, heterogeneity was substantial (*I* ^2^ = 92%), and the 95% prediction interval [−2.43 to 1.15] crossed the line of no effect, suggesting that VR efficacy varies significantly depending on the clinical context.

To investigate this heterogeneity, a subgroup analysis by procedure type was conducted. This revealed distinct patterns of efficacy (subgroup difference *p* < 0.0001) (Figure S4). For episiotomy repair, the use of VR demonstrated a large, consistent effect with zero heterogeneity (*κ* = 3, *n* = 194, SMD −1.40; 95% CI: −1.71 to −1.08, *I* ^2^ = 0%). For hysteroscopic procedures, there is a moderate, significant reduction in pain scores (*κ* = 12, *n* = 1140, SMD −0.47; 95% CI: −0.82 to −0.11), though heterogeneity remained high (*I* ^2^ = 88%). For IUD insertion, the results were inconsistent and not statistically significant with very high heterogeneity (*κ* = 5, *n* = 518, SMD −0.69; 95% CI: −1.73 to 0.34, *I* ^2^ = 96%). For the rest of the subgroup (induction of labour using extra‐amniotic balloon catheter, external cephalic version, colposcopy, amniocentesis, manual vacuum aspiration, intrauterine insemination, oocyte retrieval), there was a significant reduction in pain score (*κ* = 9, *n* = 863, SMD −0.61; 95% CI: −1.37 to 0.15).

### 8.5 Anxiety

Twenty five randomised‐controlled trials reported on anxiety levels as outcome. The impact of VR on anxiety followed a similar pattern to pain outcomes.

#### 8.5.1 Labour

In labouring women (*κ* = 8, *n* = 825), VR demonstrated a large reduction in anxiety levels (SMD −1.13; 95% CI: −1.80 to −0.45) (Figure S5). Similar to pain outcomes, the effect appeared stronger in the late stage of labour (SMD −1.34) compared to the early stage (SMD −0.84), supporting a dose–response relationship where VR becomes more effective as symptom intensity increases (Figure S6). The prediction interval [−3.48 to 1.23] included the null value, reflecting high between‐study variability (*I* ^2^ = 93%).

#### 8.5.2 Minor Procedures

For minor procedures (κ = 17, *n* = 1152), VR significantly reduced anxiety (SMD −0.74; 95% CI: −1.18 to −0.29) (Figure S7). Heterogeneity was consistently high (*I* ^2^ = 94). Subgroup analysis indicated that while VR significantly reduced anxiety during hysteroscopy (κ = 6, *n* = 550, SMD −0.50; 95% CI: −0.94 to −0.05), the effect sizes in other minor procedures were larger but highly variable (SMD −0.86; 95% CI: −1.51 to −0.22, (*I* ^2^ = 95%) (Figure S8)).

## 9 Discussion

This systematic review and meta‐analysis of 49 RCTs (*n* = 5355) represents the most comprehensive evaluation of virtual reality technology in obstetrics and gynaecology to date. Our analysis demonstrates that VR significantly reduces both pain and anxiety compared to standard care. However, the efficacy is not uniform; the magnitude of benefit depends heavily on the clinical context. The reduction in pain was most pronounced and consistent in women undergoing labour, whereas results for minor gynaecological procedures were highly variable. Additionally, VR showed a robust anxiolytic effect across all settings, often exceeding its analgesic effect size.

The experience of pain is a complex phenomenon, which simultaneously occurs on cognitive, emotional, and behavioural levels and is influenced by many factors. VR is hypothesized to reduce pain through distraction, a nonpharmacologic attentional mechanism. By providing an immersive virtual environment, the technology occupies the user's limited attentional capacity, thereby reducing the brain's ability to process incoming nociceptive signals [61]. The reduction in pain scores was observed across evaluated procedures in obstetrics and gynaecology, participant age groups, and trial designs, which increases the generalisability of our findings. Our subgroup analysis suggests that the nature of the pain determines VR's success. In the labour subgroup, VR consistently reduced pain intensity by an SMD of −0.93, corresponding to an approximate reduction of 2.2 cm on a 10 cm VAS. Importantly, the 95% prediction interval for labour excluded the null effect, giving clinicians high confidence that VR will be beneficial for future individual patients. This suggests that the high cognitive load of VR can effectively compete with the visceral, escalating pain of labour. The prolonged nature of labour allows for extended VR exposure, which may enhance the analgesic effect compared to brief minor procedures. Furthermore, labour pain has a significant psychological component; VR's ability to promote relaxation and “presence” likely contributes to its superior effectiveness in this cohort.

We observed a “dose–response” relationship where VR became numerically more effective as pain intensity increased. The effect size was larger in the transitional phase (SMD −1.15) compared to the active phase (SMD −0.77). This aligns with findings by Ozer et al. and Akin et al., who noted that VR was significantly more effective as cervical dilatation progressed [14]. However, heterogeneity remained high in the late stages of labour (*I* ^2^ = 86%). This is likely driven by the unpredictable nature of fetal descent, maternal fatigue, and variability in pushing techniques, as well as differences in VR content (e.g., passive relaxation vs. interactive content). Although the effect size was greater in the transitional stage of labour versus first stage (active phase), the transitional stage group included 6 RCTs. Future studies should focus on larger sample sizes in the second stage of labour to confirm findings and reduce potential bias. Utilising standardised VR protocols could reduce variability between trials and thus reduce heterogeneity. Also, an important consideration to take into account is that these women delivered vaginally as opposed to requiring an emergency caesarean section, which may reflect a different cohort of women.

For minor procedures, the pooled analysis showed a significant reduction in pain (SMD −0.64), but the 95% prediction interval crossed the line of no effect [−2.43 to 1.15]. Our breakdown by procedure type explains this variability. VR was highly effective and consistent (*I* ^2^ = 0%) for episiotomy repair, which is associated with somatic, surface‐level pain. In contrast, results for IUD insertion (deep visceral cramping) were inconsistent and not statistically significant. This divergence suggests that “distraction” alone may be insufficient for acute visceral cramping compared to somatic trauma, or that the short duration of such procedures does not allow sufficient time for full immersion to occur.

VR significantly reduced anxiety across all procedures. The effect size was slightly larger in reducing anxiety in labour (SMD −1.13, 95% CI −1.80 to −0.45) and minor procedures (SMD −0.74, 95% CI −1.18 to −0.29) compared to pain (SMD −0.93 and SMD −0.64 respectively). This is consistent with other studies [62, 63]. This is consistent with the finding that anxiety and pain are bidirectionally linked; anxiety lowers pain thresholds, and pain increases anxiety. VR appears to break this cycle by inducing a state of presence in a non‐threatening environment. The anxiolytic effect was particularly strong in labour (SMD −1.13), which is clinically significant given that high maternal anxiety is associated with prolonged labour and adverse fetal outcomes. Future studies should investigate the optimal VR settings for example, guided‐relaxation versus distraction‐based VR and the ideal timing and duration of VR use to maximise its calming effects. VR could be particularly useful in obstetric settings, where managing anxiety is critical for maternal and fetal wellbeing [64].

Consistent with pain scores, VR also reduced anxiety with a greater effect size in labour when compared to minor procedures. Additionally, VR had a greater effect in reducing anxiety during the late stage of labour (SMD −1.34, 95% CI −2.07 to −0.61) versus the early stage of labour (SMD −0.84, 95% CI −1.05 to −0.18). Previous studies have also found that VR significantly reduces anxiety in labour when compared to controls [14, 63]. To our knowledge there are no other existing studies that have compared the effect of VR on anxiety between the first and second stages of labour. Future research should explore the impact of VR on anxiety between the first and second stages of labour, incorporating larger sample sizes to enhance the reliability and validity of conclusions.

However, the widespread adoption of VR has been restricted by its historically high costs. Fortunately, advancements in high‐definition screens used in mobile phones have allowed VR to become a more accessible tool for pain and anxiety management [65]. Prioritising nonpharmacological pain management methods, such as VR, which are safe, cost‐effective, and free from major side effects, can improve the quality of healthcare services and increase patient satisfaction.

The main strength of our review is our comprehensive approach to evaluating the efficacy of immersive VR technology in various different O&G procedures, as opposed to previous reviews which only looked at one procedure [63, 66]. Furthermore, our review assessed both pain and anxiety as response variables in contrast to evaluating just one variable. We conducted prospective registration, implemented a comprehensive search strategy, and assessed for potential sources of bias. We included prediction intervals to estimate clinical utility, which prevents the over‐interpretation of heterogenous result. We also scrutinised heterogeneity through detailed subgrouping (e.g., separating hysteroscopy from IUDs), offering more granular guidance for clinicians than previous broad reviews.

Limitations in our study included substantial heterogeneity persisted in the minor procedure analysis, likely due to variations in control group protocols (e.g., different standard analgesia). Heterogeneity could be due to differences in the controls (standard care), as these varied in terms of analgesic agents, dosages, and administration frequencies; factors that we were unable to adjust for in our analysis. Further factors to take into account include variations in duration of procedure, patient pain tolerance, and variability in uterine contractions. Investigating these variables would require analysing individual patient data, however, individual patient data may offer limited additional insight, especially given the subjective nature of pain [67]. Secondly, as with all VR research, blinding of participants was impossible. While we restricted our sensitivity analysis to trials with low risk of bias in other domains, the subjective nature of self‐reported pain in unblinded trials carries an inherent risk of performance bias. Finally, we pooled varying types of VR delivery (immersive and non‐immersive). While both rely on attention diversion, it is possible that fully immersive, interactive environments provide superior analgesia, and future trials should aim to directly compare these modalities.

VR is a safe, effective, and non‐pharmacological option for pain and anxiety management in obstetrics and gynaecology. The evidence is strongest for its use in labour and somatic minor procedures (e.g., episiotomy repair). For deep visceral procedures like IUD insertion, efficacy is variable, and VR should be offered as an adjunct rather than a replacement for standard analgesia. Policymakers should consider integrating VR into labour ward guidelines as a cost‐effective tool to improve maternal satisfaction and reduce opioid requirements. Implementation of VR in clinical practice should consider the optimal format, duration, and timing of VR used for O&G procedures. Future research should focus on creating software tailored to specific procedures and optimising VR software content (immersive versus non‐immersive) to specific procedural needs.

## Author Contributions

B.H.A.W. conceived the idea, supervised the search and statistical analysis, and drafted the manuscript. J.J.T. drafted the protocol, conducted the search, and performed statistical analysis, edited and finalised the manuscript. F.C., A.K. and T.V. performed data extraction. M.P.R. edited the protocol, supervised the analysis, and edited the final manuscript. All authors provided critical input to the final manuscript.

## Funding

The authors received no specific funding for this study.

## Conflicts of Interest

The authors declare no conflicts of interest.

## Supporting information


**Data S1:** bjo70194‐sup‐0001‐TableS1.docx.

**Data S2:** bjo70194‐sup‐0002‐AppendixS1‐S3‐TableS1‐S2‐FigureS1‐S8.docx.

**Figure S1:** Funnel plot of standardised mean difference (SMD) for pain outcomes in minor procedure studies. Each circle represents a study, with size proportional to the inverse of its variance. Contour shading indicates significance regions to aid visual interpretation of potential small study effects. Trim‐and‐fill imputed studies are shown as open circles.

**Figure S2:** Funnel plot of standardised mean difference (SMD) for pain outcomes in labour studies. Each circle represents a study, with size proportional to the inverse of its variance. Contour shading indicates significance regions to aid visual interpretation of potential small study effects.

**Figure S3:** Forest plot showing the standardised mean difference (SMD) of VR versus control for labour pain, stratified by clinical stage. The analysis distinguishes between the Active Phase (typically 4–7 cm dilation) and the Transitional/Late Phase (typically > 7 cm dilation or second stage). The squares represent the effect estimate for each study, with the size proportional to its weight in the random‐effects analysis. Horizontal lines indicate the 95% Confidence Interval (CI). Diamonds represent the pooled effect estimate for each subgroup. A negative SMD indicates a reduction in pain favouring the VR group. CI, confidence interval; SMD, standardised mean difference.

**Figure S4:** Forest plot showing the standardised mean difference (SMD) of VR versus control for pain scores in minor gynaecological procedures, stratified by procedure type. The analysis stratifies studies into common clinical indications (e.g., Hysteroscopic procedures, IUD Insertion, Episiotomy Repair) and groups the remaining interventions under Other Minor Procedures. The squares represent the effect estimate for each study, with the size proportional to its weight in the random‐effects analysis. Horizontal lines indicate the 95% Confidence Interval (CI). Diamonds represent the pooled effect estimate for each subgroup and the overall total. A negative SMD indicates a reduction in pain favouring the VR group. CI, confidence interval; IUD, intrauterine device; SMD, standardised mean difference.

**Figure S5:** Forest plot showing the standardised mean difference (SMD) of VR versus control for anxiety score during labour.

**Figure S6:** Forest plot showing the standardised mean difference (SMD) of VR versus control for anxiety score during labour, stratified by clinical stage.

**Figure S7:** Forest plot showing the standardised mean difference (SMD) of VR versus control for anxiety score in minor procedures in obstetrics and gynaecology.

**Figure S8:** Forest plot showing the standardised mean difference (SMD) of VR versus control for anxiety scores in minor gynaecological procedures, stratified by procedure type. The analysis stratifies studies into hysteroscopic procedures and remaining minor procedures.

## Data Availability

The data that support the findings of this study are available from the corresponding author upon reasonable request.

## References

[1] R. Sinatra , “Causes and Consequences of Inadequate Management of Acute Pain,” Pain Medicine 11, no. 12 (2010): 1859–1871. 21040438 10.1111/j.1526-4637.2010.00983.x

[2] D. Glowacki , “Effective Pain Management and Improvements in Patients' Outcomes and Satisfaction,” Critical Care Nurse 35, no. 3 (2015): 33–41, quiz 43. 26033099 10.4037/ccn2015440

[3] J. J. Teh , D. J. Pascoe , S. Hafeji , et al., “Efficacy of Virtual Reality for Pain Relief in Medical Procedures: A Systematic Review and Meta‐Analysis,” BMC Medicine 22, no. 1 (2024): 64. 38355563 10.1186/s12916-024-03266-6 PMC10865524

[4] J. Dascal , M. Reid , W. W. IsHak , et al., “Virtual Reality and Medical Inpatients: A Systematic Review of Randomized, Controlled Trials,” Innovations in Clinical Neuroscience 14, no. 1–2 (2017): 14–21. 28386517 PMC5373791

[5] H. G. Hoffman , T. L. Richards , A. R. Bills , et al., “Using fMRI to Study the Neural Correlates of Virtual Reality Analgesia,” CNS Spectrums 11, no. 1 (2006): 45–51. 16400255 10.1017/s1092852900024202

[6] P. Cipresso , I. A. C. Giglioli , M. A. Raya , and G. Riva , “The Past, Present, and Future of Virtual and Augmented Reality Research: A Network and Cluster Analysis of the Literature,” Frontiers in Psychology 9 (2018): 2086. 30459681 10.3389/fpsyg.2018.02086 PMC6232426

[7] A. M. Harper , E. Wastnedge , A. Sivanathan , et al., “Virtual Reality as a Distraction Therapy in Obstetrics and Gynaecology,” BMJ Innovations 7, no. 3 (2021): 556–563.

[8] P. Amini , M. Mohammadi , R. Omani‐Samani , A. Almasi‐Hashiani , and S. Maroufizadeh , “Factors Associated With Cesarean Section in Tehran, Iran Using Multilevel Logistic Regression Model,” Osong Public Health and Research Perspectives 9, no. 2 (2018): 86–92. 29740533 10.24171/j.phrp.2018.9.2.08 PMC5935148

[9] C. Bedwell , T. Dowswell , J. P. Neilson , and T. Lavender , “The Use of Transcutaneous Electrical Nerve Stimulation (TENS) for Pain Relief in Labour: A Review of the Evidence,” Midwifery 27, no. 5 (2011): e141–e148. 20170995 10.1016/j.midw.2009.12.004

[10] C. A. Smith , K. M. Levett , C. T. Collins , H. G. Dahlen , C. C. Ee , and M. Suganuma , “Massage, Reflexology and Other Manual Methods for Pain Management in Labour,” Cochrane Database of Systematic Reviews 2018, no. 3 (2018): CD009290. 10.1002/14651858.CD009290.pub3 PMC6494169 29589380

[11] J. Bonapace , G. P. Gagné , N. Chaillet , R. Gagnon , E. Hébert , and S. Buckley , “No. 355‐Physiologic Basis of Pain in Labour and Delivery: An Evidence‐Based Approach to Its Management,” Journal of Obstetrics and Gynaecology Canada 40, no. 2 (2018): 227–245. 29447711 10.1016/j.jogc.2017.08.003

[12] M. J. Page , J. E. McKenzie , P. M. Bossuyt , et al., “The PRISMA 2020 Statement: An Updated Guideline for Reporting Systematic Reviews,” BMJ 372 (2021): n71. 33782057 10.1136/bmj.n71 PMC8005924

[13] S. L. West , G. Gartlehner , A. J. Mansfield , et al., Comparative Effectiveness Review Methods: Clinical Heterogeneity (Agency for Healthcare Research and Quality (US), 2010). 21433337

[14] B. Akin , M. Yilmaz Kocak , Z. Küçükaydın , and K. Güzel , “The Effect of Showing Images of the Foetus With the Virtual Reality Glass During Labour Process on Labour Pain, Birth Perception and Anxiety,” Journal of Clinical Nursing 30, no. 15–16 (2021): 2301–2308. 33960065 10.1111/jocn.15768

[15] M. Boyuk and N. Citak Bilgin , “Childbirth Journey Through Virtual Reality: Pain, Anxiety and Birth Perception: A Randomized Controlled Trial,” Research in Nursing & Health 48, no. 2 (2025): 179–189. 39749486 10.1002/nur.22438

[16] E. G. Carus , N. Albayrak , H. M. Bildirici , and S. G. Ozmen , “Immersive Virtual Reality on Childbirth Experience for Women: A Randomized Controlled Trial,” BMC Pregnancy and Childbirth 22, no. 1 (2022): 354. 35461248 10.1186/s12884-022-04598-y PMC9034564

[17] S. D. Cowles , T. Norton , T. Quiner , K. Hannaford , and M. Foley , “806: Virtual Reality May Decrease Pain During Labor,” American Journal of Obstetrics and Gynecology 220, no. 1 (2019): S527–S528.

[18] A. Ebrahimian , R. R. Bilandi , M. R. R. Bilandī , and Z. Sabzeh , “Comparison of the Effectiveness of Virtual Reality and Chewing Mint Gum on Labor Pain and Anxiety: A Randomized Controlled Trial,” BMC Pregnancy and Childbirth 22, no. 1 (2022): 49. 35045813 10.1186/s12884-021-04359-3 PMC8772130

[19] F. Estrella‐Juarez , M. Requena‐Mullor , J. Garcia‐Gonzalez , A. Lopez‐Villen , and R. Alarcon‐Rodriguez , “Effect of Virtual Reality and Music Therapy on the Physiologic Parameters of Pregnant Women and Fetuses and on Anxiety Levels: A Randomized Controlled Trial,” Journal of Midwifery & Women's Health 68, no. 1 (2023): 35–43. 10.1111/jmwh.13413 36383473

[20] D. P. Frey , M. E. Bauer , C. L. Bell , et al., “Virtual Reality Analgesia in Labor: The VRAIL Pilot Study‐A Preliminary Randomized Controlled Trial Suggesting Benefit of Immersive Virtual Reality Analgesia in Unmedicated Laboring Women,” Anesthesia and Analgesia 128, no. 6 (2019): e93–e96. 31094789 10.1213/ANE.0000000000003649

[21] E. Y. Gür and S. E. Apay , “The Effect of Cognitive Behavioral Techniques Using Virtual Reality on Birth Pain: A Randomized Controlled Trial,” Midwifery 91 (2020): 102856. 33478718 10.1016/j.midw.2020.102856

[22] N. Mahalan and M. V. Smitha , “Effect of Audio‐Visual Therapy on Pain and Anxiety in Labor: A Randomized Controlled Trial,” European Journal of Obstetrics & Gynecology and Reproductive Biology: X 20 (2023): 100240. 37771959 10.1016/j.eurox.2023.100240 PMC10522975

[23] L. Massov , B. Robinson , E. Rodriguez‐Ramirez , and R. Maude , “Virtual Reality Is Beneficial in Decreasing Pain in Labouring Women: A Preliminary Study,” Australian & New Zealand Journal of Obstetrics & Gynaecology 63, no. 2 (2023): 193–197. 35880315 10.1111/ajo.13591

[24] H. Mohammadi , J. Rasti , and E. Ebrahimi , “Virtual Reality, Fear of Pain and Labor Pain Intensity: A Randomized Controlled Trial,” Anesthesiology and Pain Medicine 13, no. 1 (2023): e130387. 37489168 10.5812/aapm-130387 PMC10363358

[25] N. Momenyan , A. A. Safaei , S. Hantoushzadeh , N. Momenyan , A. A. Safaei , and S. Hantoushzadeh , “Immersive Virtual Reality Analgesia in Un‐Medicated Laboring Women (During Stage 1 and 2): A Randomized Controlled Trial,” Clinical and Experimental Obstetrics & Gynecology 48, no. 1 (2021): 110–116.

[26] Z. Sunay and T. Uçar , “The Effects of Game‐Based Virtual Reality Application on Labor Pain and Childbirth Satisfaction in Primiparous Pregnant Women: A Randomized Controlled Study,” Health Care for Women International 46, no. 7 (2025): 741–760. 40576304 10.1080/07399332.2025.2522767

[27] M. S. Wong , B. M. R. Spiegel , and K. D. Gregory , “Virtual Reality Reduces Pain in Laboring Women: A Randomized Controlled Trial,” American Journal of Perinatology 38, no. 1 (2021): e167–e172. 32485759 10.1055/s-0040-1708851

[28] J. Xie and Q. Zeng , “Application of Virtual Reality Technology Combined With Moderate Perineal Protection in Natural Childbirth,” Ginekologia Polska 94, no. 12 (2023): 978–983. 36448347 10.5603/GP.a2022.0134

[29] M. Gökduman Keleş and S. Ö. Altinkaya , “The Effect of Virtual Reality Accompanied by Music on Women's Perceived Pain, Postpartum Comfort During Episiotomy Repair: A Randomized Controlled Trial,” Pain Management Nursing 26, no. 4 (2025): e398–e404. 40082095 10.1016/j.pmn.2025.02.004

[30] N. JahaniShoorab , S. Ebrahimzadeh Zagami , A. Nahvi , et al., “The Effect of Virtual Reality on Pain in Primiparity Women During Episiotomy Repair: A Randomize Clinical Trial,” Iranian Journal of Medical Sciences 40, no. 3 (2015): 219–224. 25999621 PMC4430883

[31] A. Şolt Kırca , N. Güdücü , and B. İkiz , “The Effect of Virtual Glasses Application on Pain and Anxiety During Episiotomy Repair: Randomized Controlled Trial,” Pain Management Nursing 24, no. 5 (2023): e123–e130. 37455184 10.1016/j.pmn.2023.06.004

[32] I. Kleiner , L. Mor , M. Friedman , et al., “The Use of Virtual Reality During Extra‐Amniotic Balloon Insertion for Pain and Anxiety Relief—A Randomized Controlled Trial,” American Journal of Obstetrics & Gynecology MFM 6, no. 1 (2024): 101222. 37951577 10.1016/j.ajogmf.2023.101222

[33] S. A. Almedhesh , W. T. Elgzar , H. A. Ibrahim , and H. A. Osman , “The Effect of Virtual Reality on Anxiety, Stress, and Hemodynamic Parameters During Cesarean Section,” Saudi Medical Journal 43, no. 4 (2022): 360–369. 35414614 10.15537/smj.2022.43.4.20210921 PMC9998068

[34] Y. Xu , Y. Shou , Y. Li , et al., “Virtual Reality Treatment Could Reduce Anxiety for Women Undergoing Cesarean Section With Spinal Anesthesia: A Randomized Controlled Trial,” Archives of Gynecology and Obstetrics 310, no. 3 (2024): 1509–1516. 38795137 10.1007/s00404-024-07556-5

[35] V. Smith , R. R. Warty , R. Kashyap , et al., “A Randomised Controlled Trial to Assess the Feasibility of Utilising Virtual Reality to Facilitate Analgesia During External Cephalic Version,” Scientific Reports 10, no. 1 (2020): 3141. 32081989 10.1038/s41598-020-60040-3 PMC7035335

[36] E. Brunn , M. Cheney , N. Hazen , V. Morozov , and J. K. Robinson , “Virtual‐Reality Effects on Acute Pain During Office Hysteroscopy: A Randomized Controlled Trial,” Journal of Gynecologic Surgery 38, no. 3 (2022): 214–220.

[37] N. Deo , K. Khan , J. Mak , et al., “Virtual Reality for Acute Pain in Outpatient Hysteroscopy: A Randomised Controlled Trial,” BJOG: An International Journal of Obstetrics & Gynaecology 128, no. 1 (2021): 87–95. 32575151 10.1111/1471-0528.16377

[38] J. Estadella Tarriel , J. Perelló Capó , M. Simó González , et al., “Effectiveness of Virtual Reality in Reducing Pain and Stress During Office Hysteroscopy: A Randomized Controlled Trial,” Healthcare (Basel, Switzerland) 13, no. 2 (2025): 131. 39857158 10.3390/healthcare13020131 PMC11765363

[39] Y. Fouks , G. Kern , A. Cohen , et al., “A Virtual Reality System for Pain and Anxiety Management During Outpatient Hysteroscopy—A Randomized Control Trial,” European Journal of Pain 26, no. 3 (2022): 600–609. 34748679 10.1002/ejp.1882

[40] R. Gamal , A. Zidan , W. Shawky , et al., “Virtual Reality for Pain Relief During Office Hysteroscopy: A Randomized Controlled Trial,” Obstetrics & Gynecology Science 68, no. 5 (2025): 424–432. 40997934 10.5468/ogs.24334 PMC12463480

[41] A. Olloqui , A. Tejerizo‐Garcia , C. Guillen , et al., “Virtual Reality for Pain Management and Patient Satisfaction During Outpatient Hysteroscopy: A Randomised Controlled Trial,” BJOG: An International Journal of Obstetrics & Gynaecology 132, no. 10 (2025): 1416–1425. 40400089 10.1111/1471-0528.18228

[42] J. A. Pelazas‐Hernández , D. Varillas‐Delgado , T. González‐Casado , et al., “The Effect of Virtual Reality on the Reduction of Pain in Women With an Indication for Outpatient Diagnostic Hysteroscopy: A Randomized Controlled Trial,” Journal of Clinical Medicine 12, no. 11 (2023): 3645. 37297840 10.3390/jcm12113645 PMC10254027

[43] V. Schutyser , R. Buyl , M. D. Vos , H. Tournaye , and C. Blockeel , “P–750 Clinical Efficacy of Virtual Reality for Acute Pain and Anxiety Management During Outpatient Hysteroscopy and Endometrial Biopsy in Subfertile Patients,” Human Reproduction 36, no. Supplement_1 (2021): deab130.749.

[44] T. Sewell , Y. Fung , A. Al‐Kufaishi , K. Clifford , and S. Quinn , “Does Virtual Reality Technology Reduce Pain and Anxiety During Outpatient Hysteroscopy? A Randomised Controlled Trial,” BJOG: An International Journal of Obstetrics & Gynaecology 130, no. 12 (2023): 1466–1472. 37218438 10.1111/1471-0528.17550

[45] B. Zizolfi , V. Foreste , M. G. Trinchillo , et al., “The Impact of Virtual Reality Technology in the Era of See & Treat Hysteroscopy: A Randomised Controlled Trial,” Facts, Views & Vision in ObGyn 17, no. 2 (2025): 121–129. 10.52054/FVVO.2025.48 PMC12233116 40546021

[46] I. Benazzouz , C. Bouhnik , A. Chapron , M. Esvan , V. Lavoué , and T. Brun , “Effects of Virtual Reality on Pain During Intrauterine Device Insertions: A Randomized Controlled Trial,” Journal of Gynecology Obstetrics and Human Reproduction 53, no. 1 (2024): 102706. 38013014 10.1016/j.jogoh.2023.102706

[47] S. Dumont , A. S. Page , K. Dewilde , et al., “Virtual Reality Simulation in Reducing Discomfort and Pain During Intrauterine Device Insertion: A Randomized Controlled Trial,” Contraception 148 (2025): 110939. 40348317 10.1016/j.contraception.2025.110939

[48] C. Higgins , C. Zecena Morales , J. Hocking , et al., “Virtual Reality for Analgesia During Intrauterine Device Insertion: Randomized Controlled Trial,” JMIR Serious Games 13 (2025): e72917. 40923352 10.2196/72917 PMC12416870

[49] T. Öz and N. Demirci , “The Effect of Virtual Reality Glasses Applied During Intrauterine Device Insertion on Pain, Anxiety and Satisfaction: Randomized Controlled Study,” Scottish Medical Journal 69, no. 2 (2024): 37–44. 38449359 10.1177/00369330241234688

[50] E. Toker and M. Gökduman Keleş , “The Impact of Virtual Reality With Relaxation Music and Distraction Cards on Pain, Anxiety, and Satisfaction Levels of Women With an Intrauterine Device: A Randomized Controlled Trial,” Journal of Midwifery & Women's Health 17 (2026): 104–112. 10.1111/jmwh.70048 41235692

[51] N. Baltaci , S. Bal , E. Koç , and E. K. Edis , “Effects of Virtual Reality and Nature Sounds on Pain and Anxiety During Hysterosalpingography: A Randomized Controlled Trial,” Revista Da Associacao Medica Brasileira 70, no. 7 (2024): e20231599. 39166658 10.1590/1806-9282.20231599 PMC11329239

[52] K. Rosielle , A. P. van Haaps , E. A. M. Kuijper , et al., “No Pain Relief by Virtual Reality During Hysterosalpingography (HSG): Results From a Randomized Controlled Trial,” Human Reproduction (Oxford, England) 39, no. 9 (2024): 1987–1995. 38863305 10.1093/humrep/deae133 PMC11373382

[53] N. Y. Sezer , M. N. Aker , İ. M. Gönenç , Ş. Topuz , and Y. E. Şükür , “The Effect of Virtual Reality on Women's Perceived Pain, Fear, Anxiety, and Views About the Procedure During Hysterosalpingography: A Randomized Controlled Trial,” European Journal of Obstetrics, Gynecology, and Reproductive Biology 286 (2023): 5–9. 37156133 10.1016/j.ejogrb.2023.04.028

[54] C. Chinanuwatwong , K. Kongsomboon , and T. Hanprasertpong , “Best Maternal Point of Visual Focusing for Minimizing Pain and Anxiety During Second Trimester Genetic Amniocentesis: A Randomized Trial,” BMC Pregnancy and Childbirth 25 (2025): 1353. 41299297 10.1186/s12884-025-08265-w PMC12750950

[55] Y. Melcer , R. Maymon , M. Gal‐Kochav , et al., “Analgesic Efficacy of Virtual Reality for Acute Pain in Amniocentesis: A Randomized Controlled Trial,” European Journal of Obstetrics, Gynecology, and Reproductive Biology 261 (2021): 134–138. 33932684 10.1016/j.ejogrb.2021.04.024

[56] J. M. Hecken , P. Halagiera , S. Rehman , C. B. Tempfer , and G. A. Rezniczek , “Virtual Reality for Anxiety Reduction in Women Undergoing Colposcopy: A Randomized Controlled Trial,” Journal of Lower Genital Tract Disease 27, no. 3 (2023): 223–229. 37166026 10.1097/LGT.0000000000000745

[57] M. Dviri , L. M. Haham , J. B. M. Friedler , et al., “The Use of Virtual Reality Technology in Infertile Women Undergoing In Vitro Fertilization‐Embryo Transfer: A Randomized Controlled Trial,” Fertility and Sterility 114, no. 3 (2020): e223–e224.

[58] C. S. M. Ng , P. Y. W. Tong , E. Wong , et al., “Randomized Controlled Study on the Use of Virtual Reality for Pain Relief in Oocyte Retrieval Under Transvaginal Ultrasound Guidance Using Paracervical Block and Conscious Sedation,” Human Reproduction (Oxford, England) 40, no. 12 (2025): 2310–2317. 40974211 10.1093/humrep/deaf188 PMC12675416

[59] S. Bal , N. Karakaya , E. Koç , and D. Güven , “The Effect of Virtual Reality (VR) Glasses and Therapeutic Touch (TT) on Pain, Anxiety, and Patient Satisfaction During Intrauterine Insemination (IUI) Compared to Standard Care: A Single‐Blind, Randomized Controlled Trial,” BMC Pregnancy and Childbirth 25, no. 1 (2025): 361. 40148795 10.1186/s12884-025-07435-0 PMC11948925

[60] N. S. Sibal , I. Sibal , H. Z. Aksoy , H. R. Aydin , Y. Özoran , and C. A. Sekerci , “The Effect of Virtual Reality Headset Use on Anxiety Levels During Urodynamic Testing: A Prospective, Randomized Controlled Study,” Urology 206 (2025): 48–55. 40780518 10.1016/j.urology.2025.08.002

[61] L. Kjeldgaard Pedersen , L. Y. V. Fisker , J. D. Rölfing , et al., “Virtual Reality Increases Pressure Pain Threshold and Lowers Anxiety in Children Compared With Control and Non‐Immersive Control—A Randomized, Crossover Trial,” European Journal of Pain 27, no. 7 (2023): 805–815. 36897663 10.1002/ejp.2108

[62] N. Cohen , L. A. Nasra , M. Paz , Y. Kaufman , O. Lavie , and A. Zilberlicht , “Pain and Anxiety Management With Virtual Reality for Office Hysteroscopy: Systemic Review and Meta‐Analysis,” Archives of Gynecology and Obstetrics 309, no. 4 (2024): 1127–1134. 37917158 10.1007/s00404-023-07261-9

[63] E. Özer , Y. Ç. Şen , S. Canlı , and G. Güvenç , “Effects of Virtual Reality Interventions on the Parameters of Normal Labor: A Systematic Review and Meta‐Analysis of Randomized Controlled Trials. A Meta‐Analysis of Virtual Reality Interventions on the Parameters of Normal Labor,” Pain Management Nursing 25, no. 1 (2024): 93–99. 37880013 10.1016/j.pmn.2023.09.012

[64] L. P. Hulsbosch , I. Nyklíček , E. S. Potharst , M. Meems , M. G. B. M. Boekhorst , and V. J. M. Pop , “Online Mindfulness‐Based Intervention for Women With Pregnancy Distress: Design of a Randomized Controlled Trial,” BMC Pregnancy and Childbirth 20, no. 1 (2020): 159. 32169030 10.1186/s12884-020-2843-0 PMC7069182

[65] H. G. Hoffman , E. J. Seibel , T. L. Richards , T. A. Furness , D. R. Patterson , and S. R. Sharar , “Virtual Reality Helmet Display Quality Influences the Magnitude of Virtual Reality Analgesia,” Journal of Pain 7, no. 11 (2006): 843–850. 17074626 10.1016/j.jpain.2006.04.006

[66] N. Xu , S. Chen , Y. Liu , Y. Jing , and P. Gu , “The Effects of Virtual Reality in Maternal Delivery: Systematic Review and Meta‐Analysis,” JMIR Serious Games 10, no. 4 (2022): e36695. 36416881 10.2196/36695 PMC9730208

[67] E. Rogozińska , N. Marlin , S. Thangaratinam , K. S. Khan , and J. Zamora , “Meta‐Analysis Using Individual Participant Data From Randomised Trials: Opportunities and Limitations Created by Access to Raw Data,” Evidence‐Based Medicine 22, no. 5 (2017): 157–162. 28818966 10.1136/ebmed-2017-110775

