# Supplementary material for: Effectiveness of Virtual Reality for Pain Relief in Procedures Related to Obstetrics and Gynaecology: A Systematic Review and Meta‐Analysis of Randomised Controlled Trials
Source: BJOG. 2026 Feb 24;133(7):1358–72. doi: 10.1111/1471-0528.70194 (PMC13143552; doi:10.1111/1471-0528.70194)
Supplement: Supplementary file 1 — Data S1: bjo70194‐sup‐0001‐TableS1.docx. [file BJO-133-1358-s002.docx]

**Supplementary** **Table 1:** Characteristics of randomised trials evaluating the effectiveness of virtual reality for pain and anxiety in obstetrics and gynaecology

FPS-R, Faces Pain Scale-Revised; GRS, Graphic Rating Scale; NRS, Numeric Rating Scale; STAI, Spielberger State-Trait Anxiety Inventory; VAS, Visual Analog Scale; VDS, Verbal Descriptor Scale;WBFS, Wong-Baker Faces Pain Rating scale

| Study | Inclusion criteria | Settings | Sample size | Medical procedure | Duration of procedure or intervention where appropriate | Outcomes measured (pain/anxiety/stress) | Pain and/or anxiety scale used | Adverse effect |
| --- | --- | --- | --- | --- | --- | --- | --- | --- |
| Amniocentesis | | | | | | | | |
| Chinanuwatwong 2025 | Singleton pregnancy 16-20 week for amniocentesis, without fetal anomaly | OP | 105 | Amniocentesis | 30-35 seconds | Pain | VAS | None reported |
| Melcer 2021 | Singleton pregnancy, obstetrically indicated mid-trimester amniocentesis | OP | 60 | Amniocentesis | Not specified | Pain, Anxiety | VAS | Well tolerated with no serious side effects, 2 removed headset due to nausea |
| Caesarean section | | | | | | | | |
| Almedhesh 2022 | Parturient, normal hearing and visual abilities, no history of anxiety or mental illness, no serious obstetric complications, no increased intraoperative risk | IP | 365 | Caesarean section | 46.88 minutes (VRintervention)  48.16  (control) | Anxiety, stress, satisfaction | Brief measure of emotional preoperative stress  (B-MEPS), Novel visual facial anxiety scale (NVFAS), and  Birth satisfaction scale-revised (BSS-R) | None reported but 4 unnamed complications during the caesarean section and 3 in recover in the VR group |
| Xu 2024 | American Society of Anesthesiologists (ASA) physical status 2, presenting for elective cesarean delivery under spinal anesthesia, no severe fetal malformations, gestational age >28 weeks, no severe motion sickness, no psychiatric disorders, no history of epilepsy or audiovisual impairment | IP | 128 | Caesarean section | The duration of the VR video was 30.5 min | Anxiety, satisfaction | VAS | no significant difference in side effects between the two groups |
| Colposcopy | | | | | | | | |
| Hecken 2023 | 18-80 years, pathological pap smear and/or persistent HPV high-risk positivity | OP | 247 | Colposcopy | VR1: 182±94 seconds  VR2: 218±128 seconds  (intervention),  204 ± 121 seconds (control) | Anxiety | STAI, STAI-T, STAI-S | Low disappointment rating  Not reported any adverse effects |
| Embryo transfer | | | | | | | | |
| Dviri 2020 | 21-45 years using own ovum, >45 years old using donor eggs, infertile, starting a frozen embryo transfer cycle | OP | 76 | Embryo transfer - IVF | 23.2 ± 14.1 minutes (VR exposure time) | Anxiety | STAI | None |
| Oocyte retrieval | | | | | | | | |
| Ng 2025 | 18-43 years undergoing bilateral oocyte retrieval, capable of providing informed consent | OP | 160 | Oocyte retrieval | 4 minutes and 20 seconds (VR), duration of retrieval 11.7 ± 5.7 minutes (VR), 10.9 ± 4.4 minutes (control) | Pain, Satisfaction | VAS | Nausea (2 in VR, 2 in control) |
| External cephalic version | | | | | | | | |
| Smith 2020 | Singleton pregnancy, ultrasound scan confirmed breech presentation | IP | 50 | External cephalic version | 623 ± 722 seconds (VR),  439 ± 752 (control) | Pain, Anxiety | 101 point numerical rating scale | 26% experienced side effect, no difference between two groups: dizziness, nausea, vomiting, tremulousness, flushing |
| Hysterosalpingography | | | | | | | | |
| Baltaci 2024 | >18 years, women who underwent HSG, could read and speak Turkish | IP | 135 | hysterosalpingography | Not specified | pain, anxiety | VAS, STAI | Not specified |
| Rosielle 2024 | Women referred for hysterosalpingography (HSG), history of surgery to the cervix, no HSG before, no known allergy to iodinated contrast, no current use of antidepressants, sedatives, or analgesics on a daily basis | OP | 134 | hysterosalpingography | Not specified | Pain, satisfaction, VR preferences | VAS | Seven patients removed the VR headset during the procedure. The reported reasons were discomfort (three patients), being nauseous (one patient), and too much pain from the procedure (three patients) |
| Sezer 2023 | Have primary infertility, no hearing or visual impairments, not having undergone an HSG before | OP | 62 | hysterosalpingography | Not specified | Anxiety, pain, fear, satisfaction, physiological parameters | VAS, VAS-fear, SAI, VAS-satisfaction | No side effects reported |
| Hysteroscopy | | | | | | | | |
| Brunn 2022 | >18 years, english speaking, with no current narcotic use, indicated for hysteroscopy | OP | 50 | Hysteroscopy | Minimal (median 293 seconds (VR), median 238 seconds (control) | Pain, satisfaction | VAS | Not specified |
| Deo 2021 | 18-70 years, no hearing or visual impairment | OP | 40 | Hysteroscopy | 3.25 minutes | Pain | NRS | Well tolerated with no serious side effects (nausea reported by 1 patient) |
| Fouks 2022 | ≥ 18 years, not having suspected infection, not using analgesia 6 hours prior to procedure | OP | 102 | Hysteroscopy | 8.1 ± 3.2 minutes (VRintervention),  7.3 ±6 6.0 (control) | Pain, anxiety | NRS | No differences in dizziness, nausea, vomiting, shivering and contraction or menstrual pain |
| Gamal 2025 | 18-60 years with no acute pelvic infection, not pregnant, no heavy uterine bleeding | OP | 50 | Hysteroscopy | 4 minutes (VR); 4.5 minutes (Control) | Pain, Satisfaction | NRS | None reported |
| Olloqui 2025 | 18-70 years with no visual or hearing impairment, no language barrier | OP | 120 | Hysteroscopy | 4.6 ± 3.3 min (VR), 5.6 ± 4.0 min (Control) | Pain, Satisfaction | VAS | Dizziness in 3 patients (2 required removal of headset) |
| Pelazas-Hernandez 2023 | 18-75 years, able to understand the study and give written informed consent, established by SEGO to perform office hysteroscopy due to heavy menstrual bleeding, suspected endometrial pathology, postmenopausal bleeding, removal of foreign body | OP | 154 | Hysteroscopy | 7.95 (±1.01) minutes (VRintervention)  ​​​​6.67min (±0.52)  (control) | Pain | VAS | 4 cases of vagal action (2.6%) - dizziness during hysteroscopy (3 in control and 1 in study group) |
| Schutyser 2021 | Subfertility requiring endometrial biopsy | OP | 48 | Hysteroscopy | Not specified | Pain, Anxiety, Stress | VAS | Not specified |
| Sewell 2023 | 18-70 years, any indication for hysteroscopy, able to give consent | OP | 83 | Hysteroscopy | 26 ± 5.8 minutes (VRintervention), 24.4 ± 6.7 minutes (control) | Pain, Anxiety, Satisfaction | NRS | 1 patient reported nausea and asked for headset to be remove prematurely, 1 patient requested sound on headset ot be turn off |
| Tarriel 2025 | >18 years, able to understand and accept the study procedures, not taking anxiolytic treatment | OP | 159 | Hysteroscopy | VR of 7 minutes | Pain, anxiety | VAS, STAI-S | Although not statistically significant, 27 patients in the control group (38.8%) and 16 in the intervention group (20,3%) experienced nausea or dizziness at some point during the procedure |
| Zizlofi 2025 | 18-70 years, able to provide consent | OP | 116 | Hysteroscopy (diagnostic and operative) | 6.9 ± 4.5 minutes (VR); 5.9 ± 3.3 (control) | Pain, Anxiety, Satisfaction | NRS | Mild nausea (2 in VR), vasovagal (1 in Control) |
| Intrauterine device (IUD) insertion | | | | | | | | |
| Benazzouz 2023 | >18 years, no pre-existing dizziness, severe facial wounds, or a history of epilepsy | OP | 100 | Intrauterine device insertion | Not specified | Pain, anxiety, satisfaction | 10cm numerical score | Not specified |
| Dumont 2025 | ≥18 years (or minor if guardian’s permission obtained), able to attend 6-week follow-up | OP | 195 | IUD insertion | Not reported | Pain | VAS (experience), NPS (pain) | Nausea (12.6% in VR vs 8% in control) |
| Higgins 2025 | ≥18 years and older able to provide written consent | OP | 70 | IUD insertion | 12 minutes (both VR and control) | Pain, Anxiety, Satisfaction | 10-cm VAS | Nausea (1 patient) |
| Oz 2024 | For IUD insertion | OP | 80 | Intrauterine device insertion | Not specified | pain, anxiety | VAS, STAI-S | Not specified |
| Toker 2025 | 18-45 years undergoing first IUD insertion, proficient in Turkish, no history of cervical surgery or diagnosed mental illness, not taking analgesics before IUD insertion | OP | 117 | IUD insertion | 15-20 minutes | Pain, Anxiety, Satisfaction | VAS, STAI-T | Not reported |
| Intrauterine insemination (IUI) | | | | | | | | |
| Bal 2025 | ≥18y with unexplained or male-factor infertility undergoing IUI. | OP | 96 | Intrauterine Insemination | 15 minutes before and 15 minutes during | Pain, Anxiety, Satisfaction | VAS, STAI | Not specified |
| Labour | | | | | | | | |
| Akin 2021 | >18 years, primiparous, 28th week of pregnancy, cephalic presentation | IP | 100 | Labour | Intervention 14.2 ± 14.9 minutes (VR) | Pain, anxiety | VAS, Perinatal Anxiety Screening Scale (PASS) | No side effects reported |
| Boyuk 2025 | 18–3 years, primiparous,  37–42 weeks of gestation, vaginal delivery planned, hospitalized in the delivery room in the latent phase, single pregnancy, head  first fetal presentation, without dystocia, without labor‐induction, without epidural anesthesia, | IP | 60 | Labour (Active and Transition) | 20 minutes (Active) + 10 minutes (Transition) | Pain, Anxiety | VAS-P, VAS-A | Discomfort in 4 patients (13.3%) e.g., nausea, sweating |
| Carus 2022 | 18-42 years, 37-41 weeks gestation, cephalic presentation, for vaginal birth | IP | 42 | Labour (early and first stage) | VR of 20 minutes in two phases each | Pain, anxiety, satisfaction | Wong-Baker Faces Pain Rating Scale, Beck Anxiety Inventory | No significant adverse events observed |
| Cowles 2019 | Not specified | IP | 20 | Labour (Active labour) | Not reported | Pain | VAS | Not specified |
| Ebrahimian 2022 | 18-35 years, 37-41 weeks gestation, singleton, cephalic presentation, low risk pregnancy, gravida 1 or 2 | IP | 93 | Labour, first stage | Intervention for 30 minutes at two time points (4-5cm and 7-8cm dilated) | Pain, anxiety | Visual analogue, Spielberger’s anxiety inventory | Not specified |
| Estrella-Juarez 2023 | ≥37 weeks gestation, low-risk pregnancy, nulliparous, singleton, spontaneous conception | IP | 343 | Non-stress test in third trimester and first stage of labour | Intervention for 20 minutes twice | anxiety | STAI | Not specified |
| Frey 2018 | Healthy, >32 weeks gestation, nulliparous, vaginal delivery, low risk pregnancy | IP | 27 | Labour, first stage | Unspecified | Pain | NRS | No adverse effect, no significant differences in nausea |
| Gur 2020 | No auditory or visual impairment, admitted for vaginal delivery, 38-40 weeks gestation, active labour (5-7 cm dilatation), no obstetric risks, not receiving oxytocin, live fetus | IP | 275 | Labour (Active phase) | 10 minutes  (length of intervention) | Pain | VAS, VRS | Not specified |
| Kleiner 2023 | Term pregnancies | IP | 145 | During mechanical induction of labour | Not specified | Pain, Anxiety | VAS, STAI-S | Not reported |
| Mahalan 2023 | P[rimigravida](https://www.sciencedirect.com/topics/medicine-and-dentistry/primigravida) term singleton pregnancies in the active phase of labor (4–8 cm), no obstetrical risks, had not received any [analgesia](https://www.sciencedirect.com/topics/nursing-and-health-professions/analgesia) or labor-enhancing drugs like oxytocin | IP | 74 | Labour | VR 572.7±274.7 minutes (VR), 546.5±214.9 minutes (control)  Control 546.49±214.91 minutes  Intervention 572.70±274.69 | Pain, anxiety | NPRS, AASPWL, PBI, Post-delivery birth satisfaction checklist | Not specified |
| Massov 2022 | ≥35 weeks gestation, active first stage of labour with pain score of >8 or 3 contractions in 10 minutes lasting approximately 60-90 seconds | IP | 14 | Labour (active first stage) | Not specified | Pain | NRS | None reported |
| Mohammadi 2023 | 18-42 years, primiparous, women in labour based on cervical examination and regular contractions, 37-41 weeks gestation, singleton, vertex presentation, no history of chronic medical conditions or pregnancy complications | IP | 130 | Labour | Not specified | Pain, Fear | Harman maternal fear questionnaire, VAS | Not specified |
| Momenyan 2021 | 18-45 years, >38 weeks gestation, low risk pregnancy, vaginal delivery, no other methods of analgesia | IP | 52 | Labour (first and second stage) | 10 minute intervention | Pain, Anxiety | NRS | Not specified |
| Sunay 2025 | Primiparous women 37-42 weeks, latent phase (<4cm) at admission, not using any pharmacological pain-reducing method | IP | 120 | Labour (Active & Transition) | Two sessions (duration based on game completion, approximately 3 minutes per game x 4 games) | Pain, satisfaction | NRS, VDS | None reported |
| Wong 2021 | ≥18 years, english speaking, nulliparous, term, having contractions at least every 5 minutes for preceding 30 minutes, pain score of 4-7 on VAS | IP | 40 | Labour, first stage | 30 minutes of intervention | Pain | VAS | 1 adverse event: emesis after 16 minutes of use, patient discontinued |
| Xie 2022 | 20-34 years, singleton, no complications in pregnancy and delivery | IP | 200 | Labour, second stage | 95 ± 10 minutes (VRintervention),  96 ± 12 (control) | Pain, Anxiety | VAS, VAS-A | Not specified |
| Episiotomy repair | | | | | | | | |
| Jahani Shoorab 2015 | Low risk pregnancy without obstetric complication, no history of mental illness, addiction, motion sickness and headaches | IP | 30 | Episiotomy repair | 11.4 ± 2.6 minutes (VRintervention),  13.6 ± 3.3 (control) | Pain, Satisfaction | NRS | Not specified |
| Keles 2025 | Primiparous women 18-35 years, vaginal delivery, mediolateral episiotomy, without 3rd or 4th degree tear | IP | 84 | Episiotomy repair | 16 minutes (VR); 15 minutes (control) | Pain | VAS | None reported |
| Kirca 2023 | 20-40 years, primiparous, singleton pregnancy, 37th-42nd gestational weeks, planned vaginal delivery, had a mediolateral episiotomy, vertex presentation, newborn APGAR score in the range of 7-10, had not utilized a non-pharmacological technique before | IP | 120 | Episiotomy | 15-25 minutes | Pain, anxiety | VAS, STAI | Not specified |
| Manual vacuum aspiration | | | | | | | | |
| McDougall 2024 | 18 – 50 years, able to give written informed consent, able to speak fluent English, no hearing or visual impairments, no history of epileptic seizures or claustrophobia | OP | 50 | manual vacuum aspiration | 8 minute VR played and then replayed depending on how long procedure took | Anxiety, pain | 11 point scale | Not specified |
| Outpatient gynaecological procedures | | | | | | | | |
| Oz 2024 | >18 years, no communication problems, able to speak Turkish, undergoing a gynecological procedure for the first time, no use of any medication that directly affects vital signs, no use of sedative agents, no history of psychiatric disease or mental perception problems, no hearing or visual impairments, no physical problems to squeeze a stress ball, undergoing outpatient gynecological procedure without sedation/anesthesia, able to give written and verbal informed consent | OP | 200 | outpatient gynaecological procedures | Not specified | Pain, anxiety, satisfaction | VAS, STAI, patient satisfaction evaluation | Not specified |
| Urodynamics | | | | | | | | |
| Sibal 2025 | 18-70 years undergoing first urodynamics | OP | 86 | Urodynamics | 30 minutes | Anxiety, Satisfaction | STAI-I, STAI-II | None reported |
