# Supplementary material for: Effectiveness of Virtual Reality for Pain Relief in Procedures Related to Obstetrics and Gynaecology: A Systematic Review and Meta‐Analysis of Randomised Controlled Trials
Source: BJOG. 2026 Feb 24;133(7):1358–72. doi: 10.1111/1471-0528.70194 (PMC13143552; doi:10.1111/1471-0528.70194)
Supplement: Supplementary file 2 — Data S2: bjo70194‐sup‐0002‐AppendixS1‐S3‐TableS1‐S2‐FigureS1‐S8.docx. [file BJO-133-1358-s003.docx]

**Supplementary materials**

**Effectiveness of virtual reality for pain relief in procedures related to obstetrics and gynaecology: A systematic review and meta-analysis of randomised controlled trials**

Jhia Jiat Teh^1^, Filipa Campos^2^, Adam Koczoski^1^, Trisha Valencia^1^, Michael P Rimmer^3,4^ , Bassel H Al Wattar^5,6^

**Appendix S1**: Literature search strategy for randomised trials evaluating effectiveness of virtual reality for pain relief in procedures related to obstetrics and gynaecology

**Appendix S2:** List of studies excluded from the review at full text screening and reason for exclusion

**Appendix S3:** List of studies excluded from the meta-analysis due to limited outcome reporting

**Table S1:** Characteristics of randomised trials evaluating the effectiveness of virtual reality for pain relief in procedures related to obstetrics and gynaecology

**Table S2:** Risk of bias across individual randomised trials included in the meta-analysis

**Figure S1**: Funnel plot of standardised mean difference (SMD) for pain outcomes in minor procedure studies. Each circle represents a study, with size proportional to the inverse of its variance. Contour shading indicates significance regions to aid visual interpretation of potential small study effects. Trim-and-fill imputed studies are shown as open circles.

**Figure S2:** Funnel plot of standardised mean difference (SMD) for pain outcomes in labour studies. Each circle represents a study, with size proportional to the inverse of its variance. Contour shading indicates significance regions to aid visual interpretation of potential small study effects.

**Figure S3:** Forest plot showing the standardised mean difference (SMD) of VR versus control for labour pain, stratified by clinical stage.

The analysis distinguishes between the Active Phase (typically 4–7 cm dilation) and the Transitional/Late Phase (typically >7 cm dilation or second stage). The squares represent the effect estimate for each study, with the size proportional to its weight in the random-effects analysis. Horizontal lines indicate the 95% Confidence Interval (CI). Diamonds represent the pooled effect estimate for each subgroup. A negative SMD indicates a reduction in pain favouring the VR group. *Abbreviations: CI, confidence interval; SMD, standardised mean difference.*

**Figure S4:** Forest plot showing the standardised mean difference (SMD) of VR versus control for pain scores in minor gynaecological procedures, stratified by procedure type. The analysis stratifies studies into common clinical indications (e.g., Hysteroscopic procedures, IUD Insertion, Episiotomy Repair) and groups the remaining interventions under Other Minor Procedures.

The squares represent the effect estimate for each study, with the size proportional to its weight in the random-effects analysis. Horizontal lines indicate the 95% Confidence Interval (CI). Diamonds represent the pooled effect estimate for each subgroup and the overall total. A negative SMD indicates a reduction in pain favouring the VR group. *Abbreviations: CI, confidence interval; IUD, intrauterine device; SMD, standardised mean difference.*

**Figure S5:** Forest plot showing the standardised mean difference (SMD) of VR versus control for anxiety score during labour.

**Figure S6:** Forest plot showing the standardised mean difference (SMD) of VR versus control for anxiety score during labour, stratified by clinical stage.

**Figure S7:** Forest plot showing the standardised mean difference (SMD) of VR versus control for anxiety score in minor procedures in obstetrics and gynaecology.

**Figure S8:** Forest plot showing the standardised mean difference (SMD) of VR versus control for anxiety scores in minor gynaecological procedures, stratified by procedure type. The analysis stratifies studies into hysteroscopic procedures and remaining minor procedures.

**Appendix S1**: Literature search strategy for randomised trials evaluating effectiveness of virtual reality for pain relief in procedures related to obstetrics and gynaecology

MEDLINE

| Line | Search Query |
| --- | --- |
| 1 | exp virtual reality/ or exp virtual reality exposure therapy/ |
| 2 | ((Simulated or augmented or mediated or mixed) adj3 (reality or world* or environment*)).tw. |
| 3 | ((Head or helmet) adj mounted).tw. |
| 4 | (Virtual or virtuality or VR or Computer interface or computer simulation or Immersi* or Interact* or Distract*).tw. |
| 5 | 1 or 2 or 3 or 4 |
| 6 | exp pain/ |
| 7 | exp anxiety/ or anxiety.mp. |
| 8 | exp patient comfort/ or discomfort.mp. |
| 9 | exp pain assessment/ |
| 10 | exp analgesia/ or analgesia.mp. |
| 11 | exp fear/ or fear.mp. |
| 12 | (pain* or anesthe* or analges* or anxious* or anxiet* or distress* or fear* or worry* or agitat* or apprehensi* or discomfort).mp. |
| 13 | 6 or 7 or 8 or 9 or 10 or 11 or 12 |
| 14 | gyn?ecolog*.mp. or exp gynecology/ |
| 15 | hysteroscopy.mp. or exp hysteroscopy/ |
| 16 | colposcopy.mp. or exp colposcopy/ |
| 17 | exp infertility therapy/ or infertility.mp. |
| 18 | exp fertilization in vitro/ or in vitro fertilisation.mp. |
| 19 | exp embryo transfer/ or embryo transfer.mp. |
| 20 | exp uterine cervix biopsy/ or cervical biopsy.mp. |
| 21 | intrauterine device.mp. or exp intrauterine contraceptive device/ |
| 22 | (dilatation and curettage).mp. |
| 23 | exp obstetric operation/ or obstetric procedure/ |
| 24 | obstetric*.mp. |
| 25 | exp labor/ or exp childbirth/ |
| 26 | labo?r.mp. |
| 27 | exp episiotomy/ or exp vaginal delivery/ or episiotomy.mp. |
| 28 | exp external cephalic version/ or exp external version/ or f?etal version.mp. |
| 29 | exp cesarean section/ or exp instrumental delivery/ |
| 30 | c?esarean.mp. |
| 31 | exp amniocentesis/ or exp prenatal diagnosis/ or amniocentesis.mp. |
| 32 | exp endometriosis/ or endometriosis.mp. |
| 33 | urodynamics/ or urodynamic*.mp. |
| 34 | exp cystoscopy/ or cystoscopy.mp. |
| 35 | 14 or 15 or 16 or 17 or 18 or 19 or 20 or 21 or 22 or 23 or 24 or 25 or 26 or 27 or 28 or 29 or 30 or 31 or 32 or 33 or 34 |
| 36 | ((randomized controlled trial or controlled clinical trial).pt. or randomized.ab. or placebo.ab. or drug therapy.fs. or randomly.ab. or trial.ab. or groups.ab.) not (exp animals/ not humans.sh.) |
| 37 | 5 and 13 and 35 and 36 |

EMBASE

| Line | Search Query |
| --- | --- |
| 1 | exp virtual reality/ |
| 2 | ((Simulated or augmented or mediated or mixed) adj3 (reality or world* or environment*)).tw. |
| 3 | ((Head or helmet) adj mounted).tw. |
| 4 | (Virtual or virtuality or VR or Computer interface or computer simulation or Immersi* or Interact* or Distract*).tw. |
| 5 | 1 or 2 or 3 or 4 |
| 6 | exp pain/ or exp pain assessment/ |
| 7 | exp anxiety/ or exp fear/ |
| 8 | exp discomfort/ or exp patient comfort/ |
| 9 | exp analgesia/ |
| 10 | (pain* or anesthe* or analges* or anxious* or anxiet* or distress* or fear* or worry* or agitat* or apprehensi* or discomfort).mp. |
| 11 | 6 or 7 or 8 or 9 or 10 |
| 12 | exp gynecology/ or gyn?ecolog*.mp. |
| 13 | exp hysteroscopy/ or exp colposcopy/ |
| 14 | exp infertility therapy/ or exp fertilization in vitro/ |
| 15 | exp embryo transfer/ or exp uterine cervix biopsy/ |
| 16 | exp intrauterine contraceptive device/ |
| 17 | exp obstetrics/ or exp obstetric procedure/ |
| 18 | exp labor/ or exp childbirth/ or labo?r.mp. |
| 19 | exp episiotomy/ or exp vaginal delivery/ |
| 20 | exp fetus version/ or exp cesarean section/ or c?esarean.mp. |
| 21 | exp instrumental delivery/ or exp amniocentesis/ |
| 22 | exp prenatal diagnosis/ or exp endometriosis/ |
| 23 | exp urodynamics/ or exp cystoscopy/ |
| 24 | or/12-23 |
| 25 | 5 and 11 and 24 |
| 26 | Randomized controlled trial/ or Controlled clinical trial/ |
| 27 | (random* or placebo* or blind* or assign*).tw. |
| 28 | 26 or 27 |
| 29 | 25 and 28 |

CENTRAL

| ID | Search Query |
| --- | --- |
| #1 | MeSH descriptor: [Virtual Reality] explode all trees |
| #2 | MeSH descriptor: [Virtual Reality Exposure Therapy] explode all trees |
| #3 | ((Simulated or augmented or mediated or mixed) near/3 (reality or world* or environment*)):ti,ab,kw |
| #4 | ((Head or helmet) next mounted):ti,ab,kw |
| #5 | (Virtual or virtuality or VR or "Computer interface" or "computer simulation" or Immersi* or Interact* or Distract*):ti,ab,kw |
| #6 | #1 or #2 or #3 or #4 or #5 |
| #7 | MeSH descriptor: [Pain] explode all trees |
| #8 | MeSH descriptor: [Anxiety] explode all trees |
| #9 | (pain* or anesthe* or analges* or anxious* or anxiet* or distress* or fear* or worry* or agitat* or apprehensi* or discomfort):ti,ab,kw |
| #10 | #7 or #8 or #9 |
| #11 | MeSH descriptor: [Obstetrics] explode all trees |
| #12 | MeSH descriptor: [Gynecology] explode all trees |
| #13 | (gynaecolog* or gynecolog* or hysteroscopy or colposcopy or infertility or "in vitro fertilisation" or "embryo transfer" or "intrauterine device" or labor or labour or birth or episiotomy or cesarean or caesarean or amniocentesis or endometriosis or cystoscopy):ti,ab,kw |
| #14 | #11 or #12 or #13 |
| #15 | #6 and #10 and #14 |

**CINAHL**

| Line | Search Query |
| --- | --- |
| S1 | (MH "Virtual Reality+") OR (MH "Computer Simulation+") |
| S2 | TI ( "virtual reality" OR VR OR "augmented reality" OR "simulated reality" OR "computer interface" ) OR AB ( "virtual reality" OR VR OR "augmented reality" OR "simulated reality" OR "computer interface" ) |
| S3 | TI ( Immersi* OR Interact* OR Distract* ) OR AB ( Immersi* OR Interact* OR Distract* ) |
| S4 | S1 OR S2 OR S3 |
| S5 | (MH "Pain+") OR (MH "Anxiety+") OR (MH "Fear+") |
| S6 | TI ( pain* OR anxi* OR fear* OR discomfort OR distress OR analges* ) OR AB ( pain* OR anxi* OR fear* OR discomfort OR distress OR analges* ) |
| S7 | S5 OR S6 |
| S8 | (MH "Obstetrics+") OR (MH "Gynecology+") OR (MH "Labor+") OR (MH "Childbirth+") |
| S9 | (MH "Hysteroscopy") OR (MH "Colposcopy") OR (MH "Intrauterine Devices") |
| S10 | TI ( gynaecolog* OR gynecolog* OR obstetric* OR labor OR labour OR birth OR c?esarean OR hysteroscopy OR amniocentesis ) OR AB ( gynaecolog* OR gynecolog* OR obstetric* OR labor OR labour OR birth OR c?esarean OR hysteroscopy OR amniocentesis ) |
| S11 | S8 OR S9 OR S10 |
| S12 | S4 AND S7 AND S11 |
| S13 | MH "Clinical Trials+" |
| S14 | PT "Randomized Controlled Trial" |
| S15 | TI ( random* or trial or placebo ) OR AB ( random* or trial or placebo ) |
| S16 | S13 OR S14 OR S15 |
| S17 | S12 AND S16 |

**Appendix S2:** List of studies excluded from the review at full text screening and reason for exclusion

| **Study** | **Reason for exclusion** |
| --- | --- |
| Asiri 2022 | Study protocol |
| Elliott 2015 | Different outcome |
| Goergen 2022 | Procedure not relevant to O&G |
| Ketsuwan 2022 | Procedure not relevant to O&G |
| Luczak 2021 | Procedure not relevant to O&G |
| Lutfi 2023 | Procedure not relevant to O&G |
| Merlot 2022 | Procedure not relevant to O&G |
| Miazga 2022 | Non-immersive VR |
| Noben 2019 | Inadequate data format |
| Payne 2022 | Inadequate data format |
| Pirad 2025 | Hypnosis technique (no control group data) |
| Reinders 2022 | Procedure not relevant to O&G |
| Sun 2023 | No control group data |
| Varnier 2021 | Inadequate data format |
| Walker 2014 | Procedure not relevant to O&G |
| Yildirim 2025 | Non-immersive VR |
| Yun 2025 | Ineligible comparator (no control group data) |

**Appendix S3:** List of studies excluded from the meta-analysis due to limited outcome reporting

| **Anxiety** | **Pain** |
| --- | --- |
| Dviri 2020 | Carus 2022 |
| Fouks 2022 | Frey 2018 |
| Frey 2018 | Schutyser 2021 |
| Hecken 2023 |  |
| Melcer 2021 |  |
| Oz 2024a |  |
| Schuyster 2021 |  |
| Varnier 2021 |  |

**Supplementary Table 1:** Characteristics of randomised trials evaluating the effectiveness of virtual reality for pain relief in procedures related to obstetrics and gynaecology **(see separate document)**

**Supplementary Table 2:** Risk of bias across individual randomised trials included in the meta-analysis

| Study | Randomisation | Adherence to intervention groups | Outcome assessment | Detection | Reporting | Overall |
| --- | --- | --- | --- | --- | --- | --- |
| Akin 2021 | Some concerns | Low | Low | Low | Low | Some concerns |
| Almedhesh 2022 | Low | Low | Low | Low | Low | Low |
| Bal 2025 | Low | Low | Low | Low | Low | Low |
| Baltaci 2024 | Low | Low | Low | Low | Low | Low |
| Benazzouz 2023 | Low | Some concerns | Some concerns | Low | Low | Some concerns |
| Boyuk 2025 | Low | Some concerns | Low | Some concerns | Low | Some concerns |
| Brunn 2022 | Low | Low | Low | Low | Some concerns | Some concerns |
| Carus 2022 | Low | Low | Low | Low | Low | Low |
| Chinanuwatwong 2025 | Low | Low | Low | Some concerns | Low | Some concerns |
| Cowles 2019 | Some concerns | Some concerns | Some concerns | Low | High | High |
| Deo 2020 | Some concerns | Low | Low | Low | Low | Low |
| Dumont 2025 | Low | Low | Low | Low | Low | Low |
| Dviri 2020 | Some concerns | Some concerns | Some concerns | Low | Some concerns | Some concerns |
| Ebrahimian 2022 | Low | Low | Low | Low | Low | Low |
| Estrella-Juarez 2022 | Low | Low | Low | Low | Low | Low |
| Fouks 2022 | Some concerns | Low | Low | Low | Low | Low |
| Frey 2018 | Low | Low | Low | Low | Low | Domain S (carryover effect):  Low /Overall:Low |
| Gamal 2025 | Low | Low | Low | Low | Low | Low |
| Gür 2020 | Low | Low | Low | Low | Low | Low |
| Hecken 2023 | Some concerns | High | Low | Some concerns | Low | Some concerns |
| Higgins 2025 | Low | Some concerns | Low Risk | Some concerns | Low | Some concerns |
| JahaniShoorab 2015 | Some concerns | Low | Some concerns | Low | Some concerns | Some concerns |
| Keles 2025 | Low | Low | Low | Low | Low | Low |
| Kirca 2023 | Low | Low | Low | Low | Low | Low |
| Kleiner 2023 | Low | Low | Low | Low | Low | Low |
| Mahalan 2023 | Low | Low | Low | Low | Low | Low |
| Massov 2022 | High | Some concerns | Low | Some concerns | Low | Domain S:  High/ Overall: High |
| McDougall 2024 | Low | Low | Low | Low | Low | Low |
| Melcer 2021 | Low | Low | Low | Low | High | Some concerns |
| Mohammadi 2023 | Some concerns | Some concerns | Low | Low | Low | Low |
| Momenyan 2021 | Low | Low | Low | Low | Low | Low |
| Ng 2025 | Low | Some concerns | Some concerns | Low | Low | Some concerns |
| Olloqui 2025 | Low | Some concerns | Low | Some concerns | Low | Some concerns |
| Oz 2024a | Low | Low | Low | Low | Low | Low |
| Oz 2024b | Low | Low | Low | Low | Low | Low |
| Pelazas-Hernandez 2023 | Low | Low | Low | Low | Low | Low |
| Rosielle 2024 | Low | Low | Low | Low | Low | Low |
| Schutyser 2021 | Some concerns | Some concerns | High | Some concerns | Some concerns | High |
| Sewell 2023 | Low | Low | Low | Low | Low | Low |
| Sezer 2023 | Some concerns | Low | Low | Low | Low | Some concerns |
| Sibal 2025 | Low | Some concerns | Low | Some concerns | Low | Some concerns |
| Smith 2020 | Low | Low | Low | Low | Low | Low |
| Sunay 2025 | Low | Some concerns | Some concerns | Some concerns | Low | Some concerns |
| Tarriel 2025 | Low | Low | Low | Low | Low | Low |
| Toker 2025 | Low | Low | Low | Low | Low | Low |
| Wong 2021 | Low | Low | Low | Low | Low | Low |
| Xie 2022 | Some concerns | Low | Low | Low | Some concerns | Some concerns |
| Xu 2024 | Low | Low | Low | Low | Low | Low |
| Zizolfi 2025 | Low | Low | Low | Low | Low | Low |

**Figure S1**: Funnel plot of standardised mean difference (SMD) for pain outcomes in minor procedure studies. Each circle represents a study, with size proportional to the inverse of its variance. Contour shading indicates significance regions to aid visual interpretation of potential small study effects. Trim-and-fill imputed studies are shown as open circles.

**Figure S2:** Funnel plot of standardised mean difference (SMD) for pain outcomes in labour studies. Each circle represents a study, with size proportional to the inverse of its variance. Contour shading indicates significance regions to aid visual interpretation of potential small study effects.

**Figure S3:** Forest plot showing the standardised mean difference (SMD) of VR versus control for labour pain, stratified by clinical stage.

The analysis distinguishes between the Active Phase (typically 4–7 cm dilation) and the Transitional/Late Phase (typically >7 cm dilation or second stage). The squares represent the effect estimate for each study, with the size proportional to its weight in the random-effects analysis. Horizontal lines indicate the 95% Confidence Interval (CI). Diamonds represent the pooled effect estimate for each subgroup. A negative SMD indicates a reduction in pain favouring the VR group. *Abbreviations: CI, confidence interval; SMD, standardised mean difference.*

**Figure S4:** Forest plot showing the standardised mean difference (SMD) of VR versus control for pain scores in minor gynaecological procedures, stratified by procedure type. The analysis stratifies studies into common clinical indications (e.g., Hysteroscopic procedures, IUD Insertion, Episiotomy Repair) and groups the remaining interventions under Other Minor Procedures.

The squares represent the effect estimate for each study, with the size proportional to its weight in the random-effects analysis. Horizontal lines indicate the 95% Confidence Interval (CI). Diamonds represent the pooled effect estimate for each subgroup and the overall total. A negative SMD indicates a reduction in pain favouring the VR group. *Abbreviations: CI, confidence interval; IUD, intrauterine device; SMD, standardised mean difference.*

**Figure S5:** Forest plot showing the standardised mean difference (SMD) of VR versus control for anxiety score during labour.

**Figure S6:** Forest plot showing the standardised mean difference (SMD) of VR versus control for anxiety score during labour, stratified by clinical stage.

**Figure S7:** Forest plot showing the standardised mean difference (SMD) of VR versus control for anxiety score in minor procedures in obstetrics and gynaecology.

**Figure S8:** Forest plot showing the standardised mean difference (SMD) of VR versus control for anxiety scores in minor gynaecological procedures, stratified by procedure type. The analysis stratifies studies into hysteroscopic procedures and remaining minor procedures.
