# Supplementary figures and images for: Effectiveness of Virtual Reality for Pain Relief in Procedures Related to Obstetrics and Gynaecology: A Systematic Review and Meta‐Analysis of Randomised Controlled Trials
Source: BJOG. 2026 Feb 24;133(7):1358–72. doi: 10.1111/1471-0528.70194 (PMC13143552; doi:10.1111/1471-0528.70194)

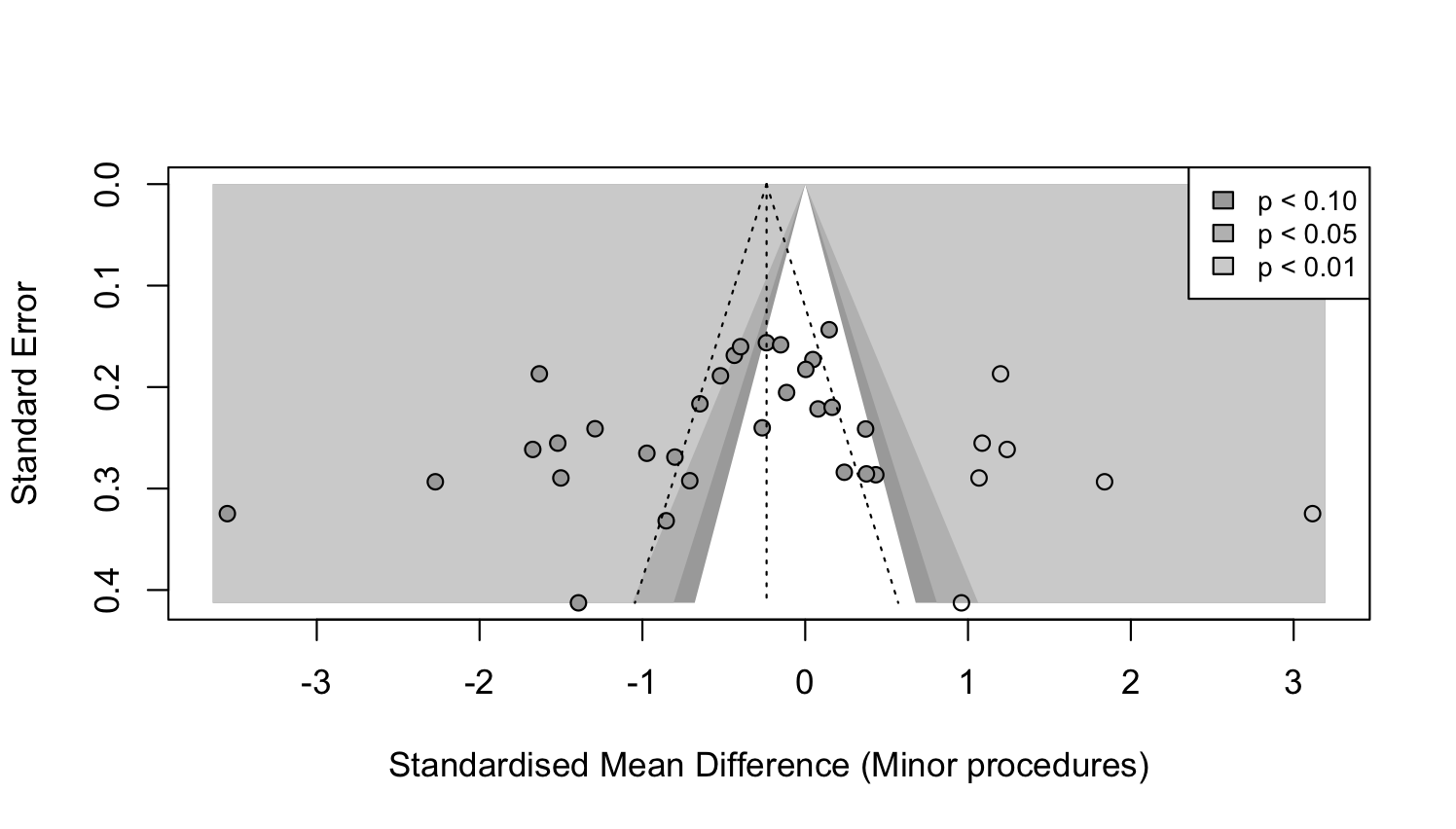

Supplement: Supplementary file 3 — Figure S1: Funnel plot of standardised mean difference (SMD) for pain outcomes in minor procedure studies. Each circle represents a study, with size proportional to the inverse of its variance. Contour shading indicates significance regions to aid visual interpretation of potential small study effects. Trim‐and‐fill imputed studies are shown as open circles. [file BJO-133-1358-s007.png]

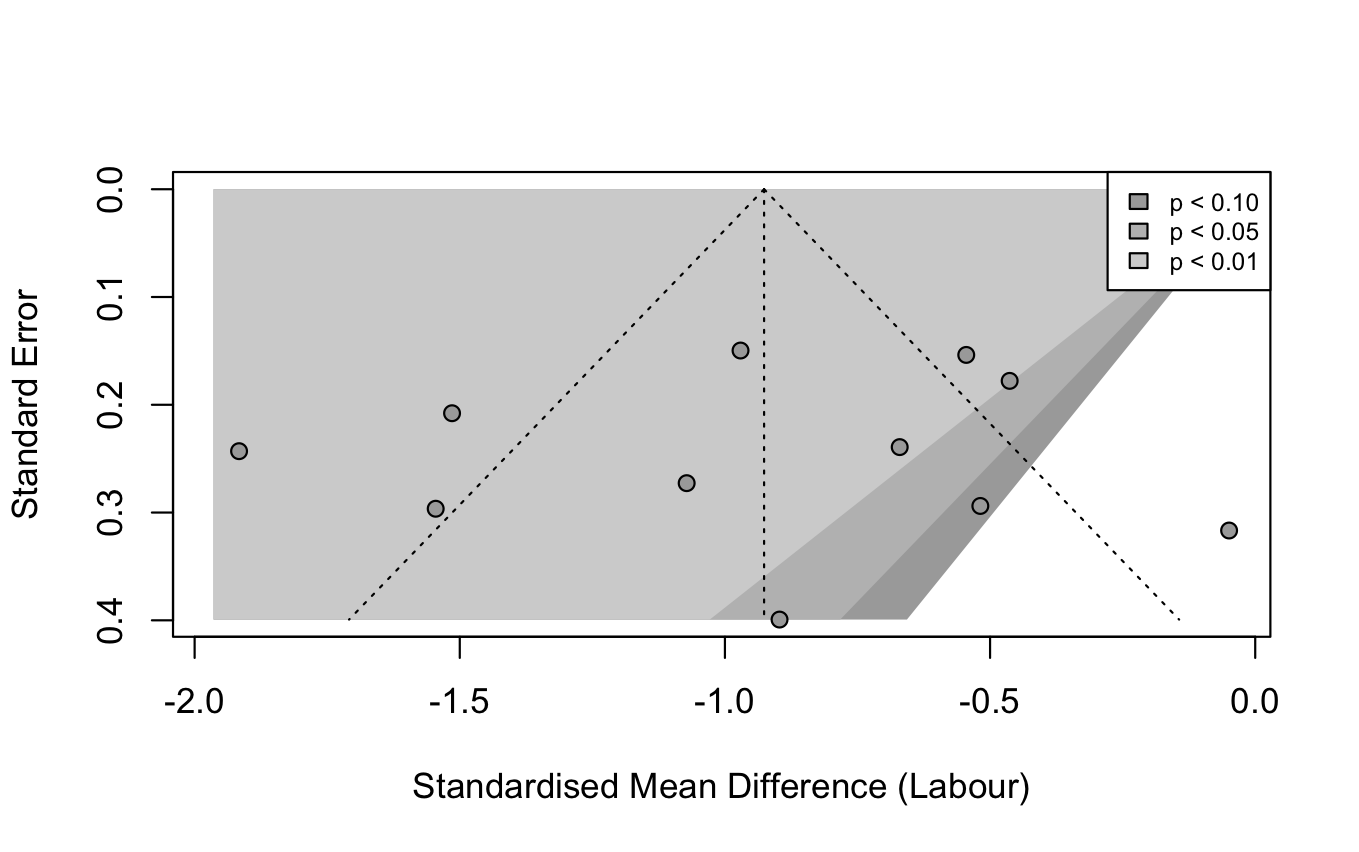

Supplement: Supplementary file 4 — Figure S2: Funnel plot of standardised mean difference (SMD) for pain outcomes in labour studies. Each circle represents a study, with size proportional to the inverse of its variance. Contour shading indicates significance regions to aid visual interpretation of potential small study effects. [file BJO-133-1358-s005.png]

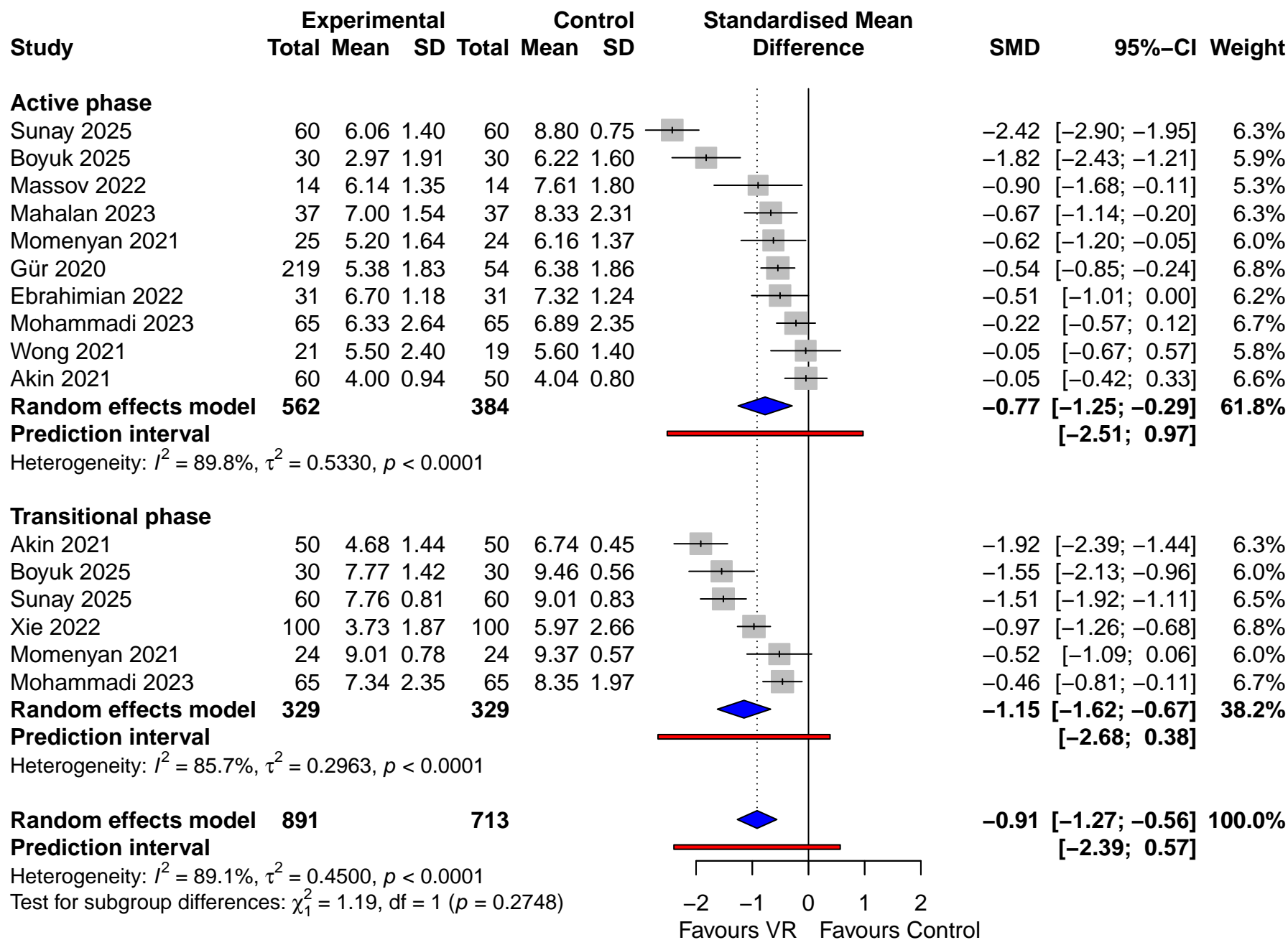

Supplement: Supplementary file 5 — Figure S3: Forest plot showing the standardised mean difference (SMD) of VR versus control for labour pain, stratified by clinical stage. The analysis distinguishes between the Active Phase (typically 4–7 cm dilation) and the Transitional/Late Phase (typically > 7 cm dilation or second stage). The squares represent the effect estimate for each study, with the size proportional to its weight in the random‐effects analysis. Horizontal lines indicate the 95% Confidence Interval (CI). Diamonds represent the pooled effect estimate for each subgroup. A negative SMD indicates a reduction in pain favouring the VR group. CI, confidence interval; SMD, standardised mean difference. [file BJO-133-1358-s008.pdf]

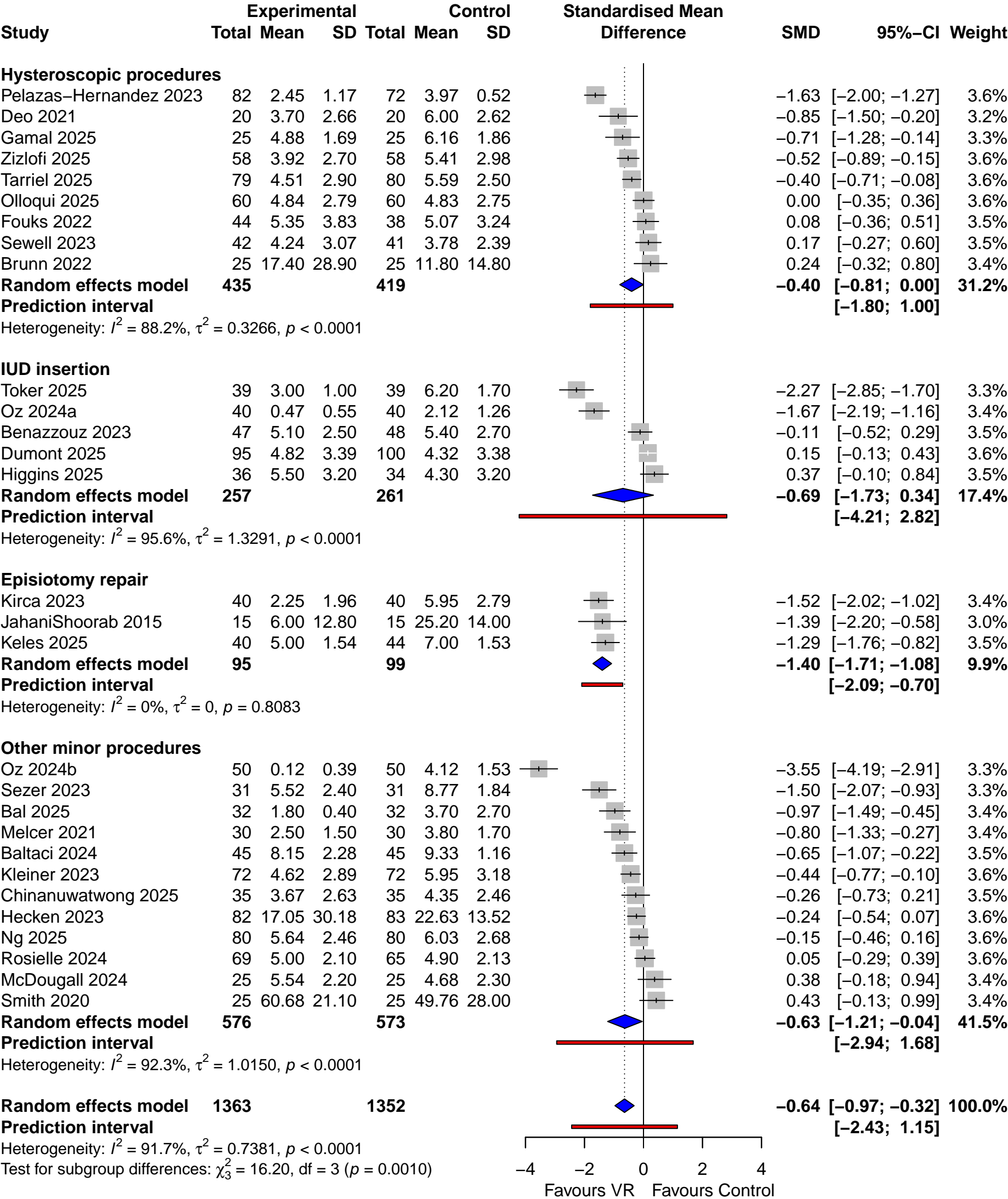

Supplement: Supplementary file 6 — Figure S4: Forest plot showing the standardised mean difference (SMD) of VR versus control for pain scores in minor gynaecological procedures, stratified by procedure type. The analysis stratifies studies into common clinical indications (e.g., Hysteroscopic procedures, IUD Insertion, Episiotomy Repair) and groups the remaining interventions under Other Minor Procedures. The squares represent the effect estimate for each study, with the size proportional to its weight in the random‐effects analysis. Horizontal lines indicate the 95% Confidence Interval (CI). Diamonds represent the pooled effect estimate for each subgroup and the overall total. A negative SMD indicates a reduction in pain favouring the VR group. CI, confidence interval; IUD, intrauterine device; SMD, standardised mean difference. [file BJO-133-1358-s009.pdf]

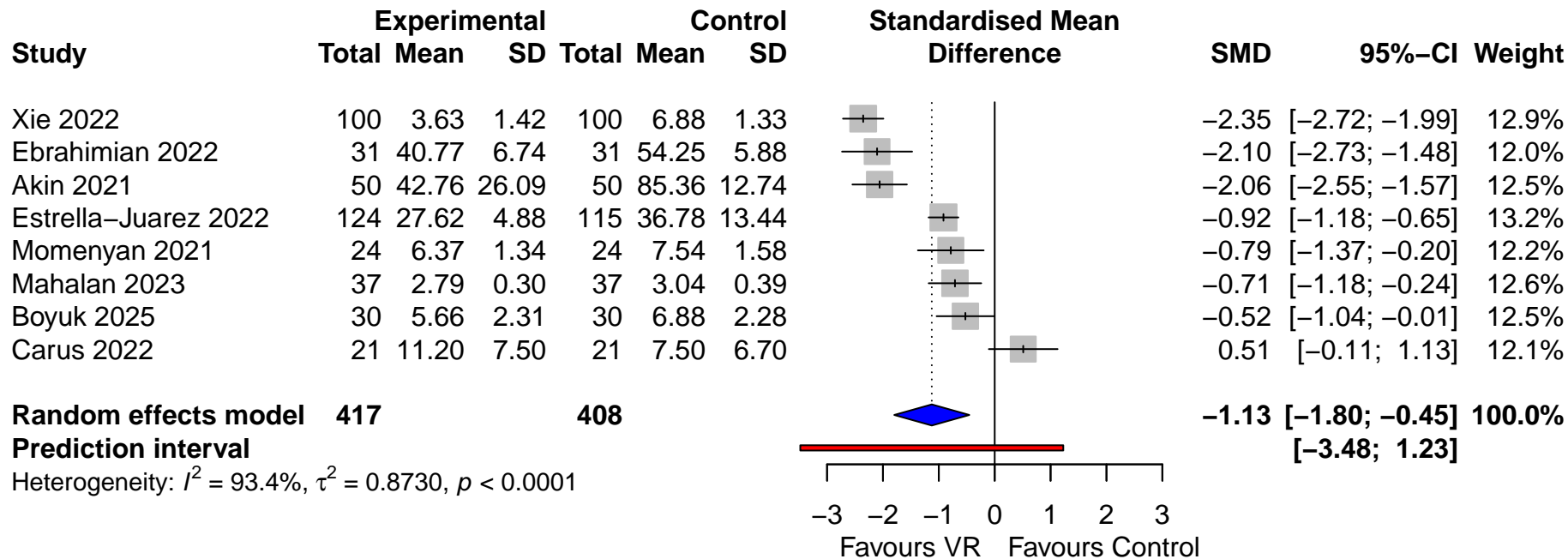

Supplement: Supplementary file 7 — Figure S5: Forest plot showing the standardised mean difference (SMD) of VR versus control for anxiety score during labour. [file BJO-133-1358-s010.pdf]

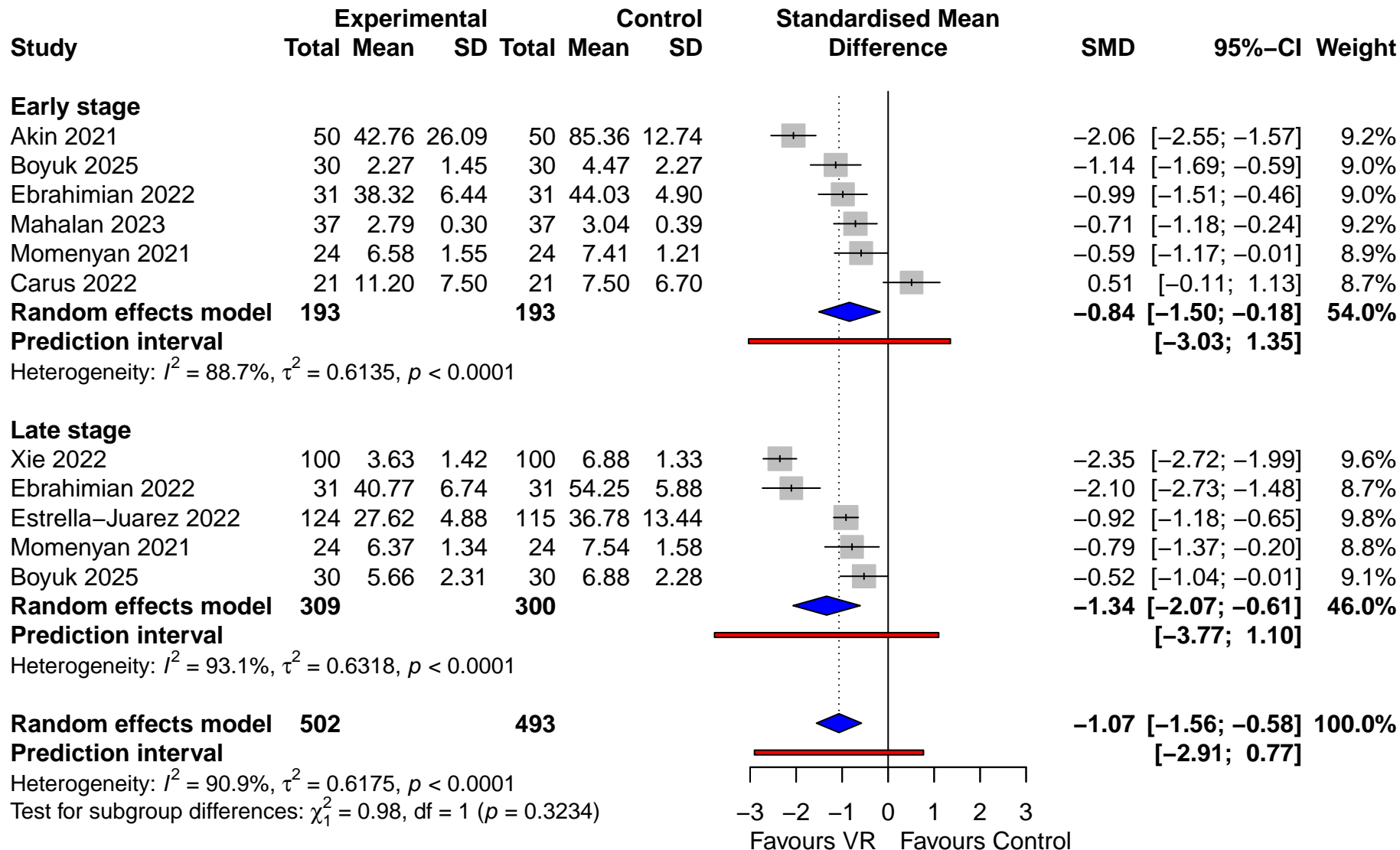

Supplement: Supplementary file 8 — Figure S6: Forest plot showing the standardised mean difference (SMD) of VR versus control for anxiety score during labour, stratified by clinical stage. [file BJO-133-1358-s006.pdf]

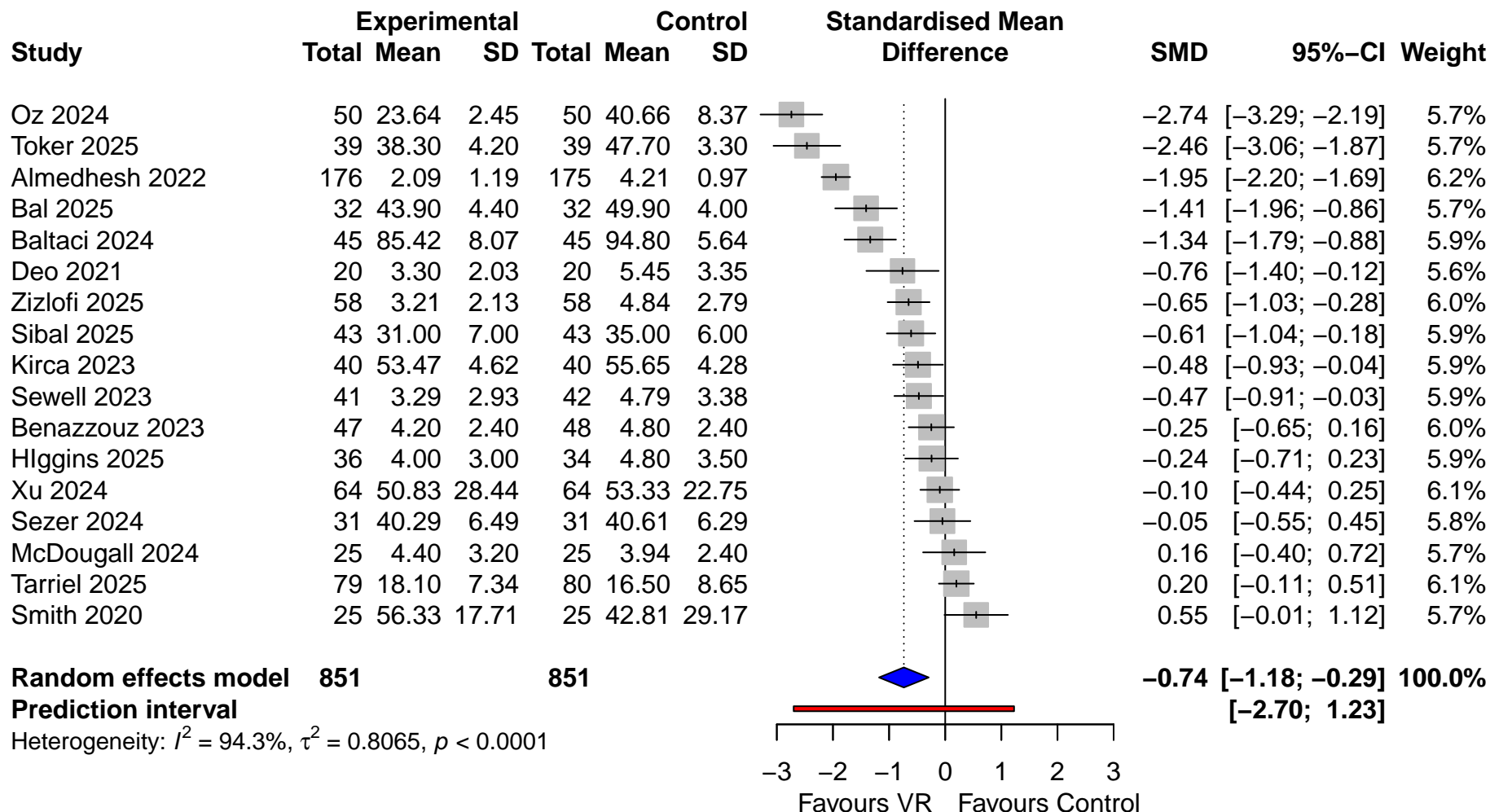

Supplement: Supplementary file 9 — Figure S7: Forest plot showing the standardised mean difference (SMD) of VR versus control for anxiety score in minor procedures in obstetrics and gynaecology. [file BJO-133-1358-s001.pdf]

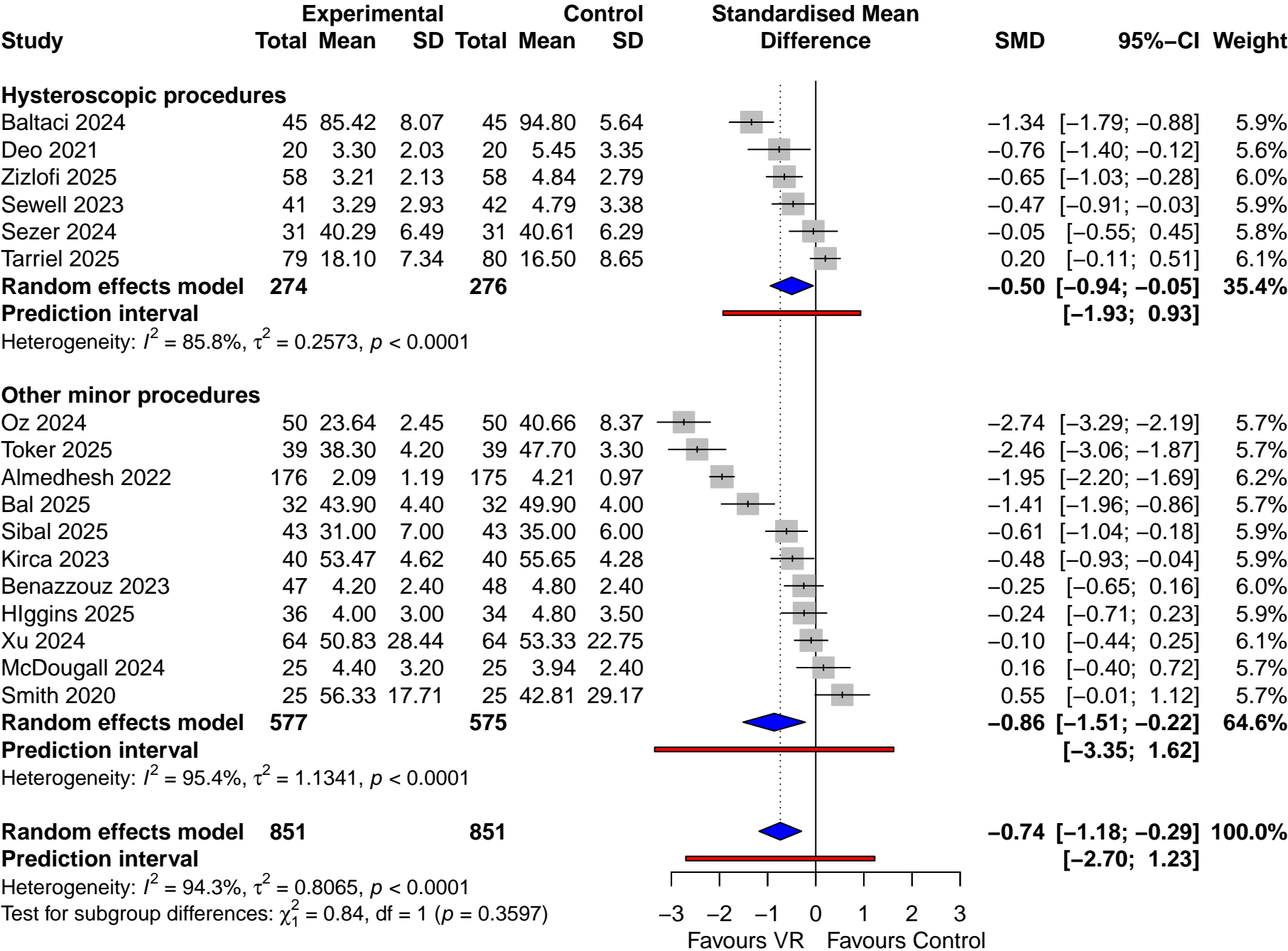

Supplement: Supplementary file 10 — Figure S8: Forest plot showing the standardised mean difference (SMD) of VR versus control for anxiety scores in minor gynaecological procedures, stratified by procedure type. The analysis stratifies studies into hysteroscopic procedures and remaining minor procedures. [file BJO-133-1358-s004.pdf]
